# Supplementary figures and images for: Insulin secretion deficits in a Prader-Willi syndrome β-cell model are associated with a concerted downregulation of multiple endoplasmic reticulum chaperones
Source: PLoS Genet. 2023 Apr 17;19(4):e1010710. doi: 10.1371/journal.pgen.1010710 (PMC10138222; doi:10.1371/journal.pgen.1010710)

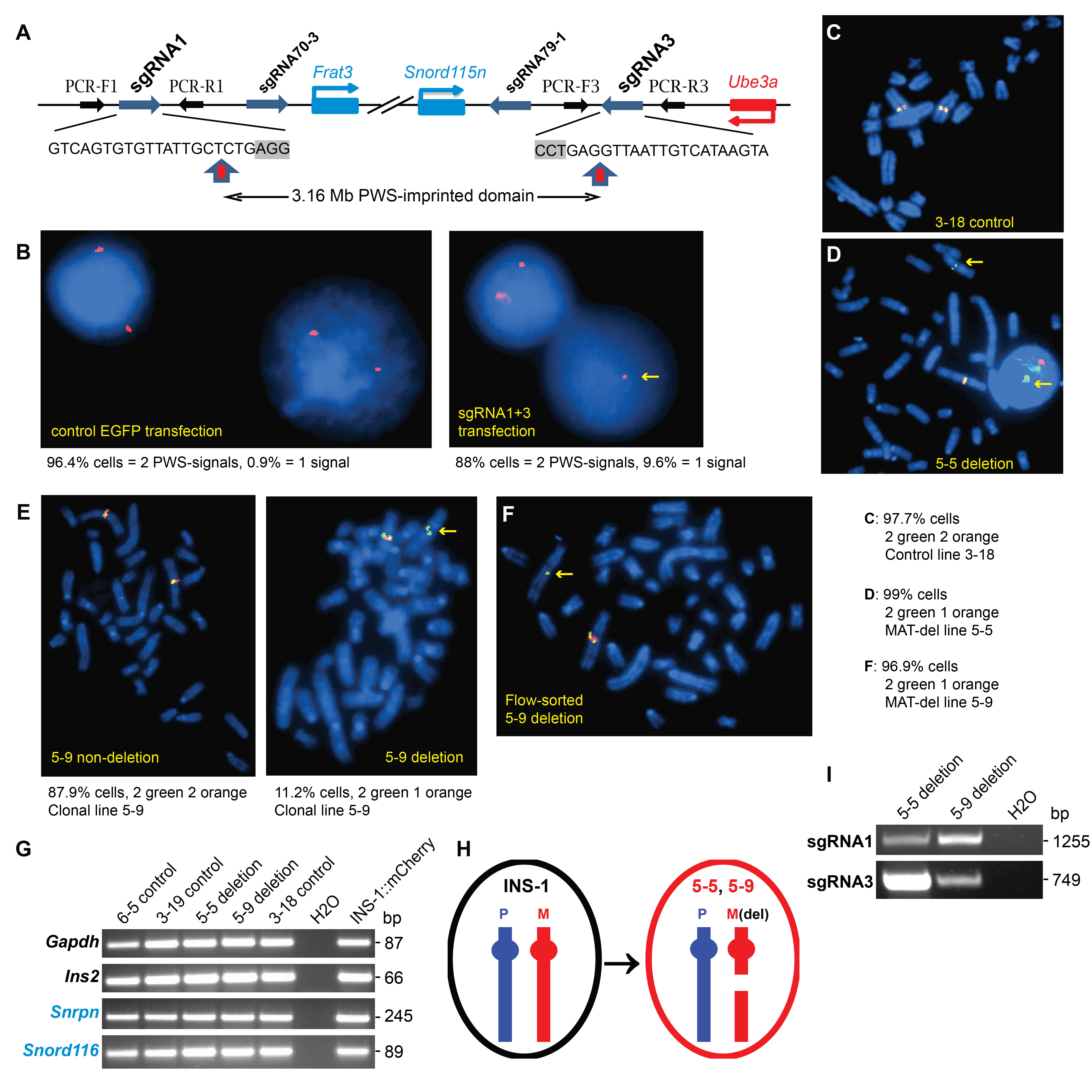

Supplement: S1 Fig — (A) Map of proximal sgRNA1 and distal sgRNA3 targeting sites. DNA sequences of the sgRNA targeting sites (blue arrows) are shown, with PAM motifs in grey highlight. Other symbols: blue boxes, proximal and distal PWS paternally-expressed genes (also see Fig 1A); red box, flanking maternally-expressed gene; black arrows, PCR primers (F, forward; R, reverse); vertical arrows, canonical double-strand break (DSB) position catalyzed by CRISPR/Cas9. (B-F) Fluorescence in situ hybridization (FISH) with rat BAC probes CH230-114P11 spanning the PWS-IC (see Methods; Fig 1A) and control CH230-2B12 (outside the PWS-domain) labeled with Orange-dUTP or Green-dUTP, respectively. Representative interphase nuclei or partial metaphases are shown. Yellow arrows, deletion of the PWS-domain. (B) 1-color FISH for parental INS-1::mCherry (INS-1) cells transfected with plasmid vectors expressing EGFP (left) or CRISPR (sgRNA1 + sgRNA3)/Cas9 (right). Most INS-1 control cells show diploid PWS BAC (orange) signals, with < 1% of cells having a single PWS-signal due to either a technical artifact (hybridization to a single allele) or to loss of a chromosome (while a small percent of cells shows increased signals due to artifact or to aneusomy). In contrast, genome editing greatly increases the percentage of cells with hemizygosity for the PWS-signal and hence a deletion for the PWS-locus. (C) Two-color FISH for control line 3–18, with most cells showing diploid signals for both BAC probes. (D) FISH for maternal (MAT)-deletion (del) line 5–5, with virtually all cells showing deletion of the PWS-locus. (E) FISH for MAT-del line 5–9 showing mosaicism, one cell population intact (and negative for the mCherry transgene) and one (mCherry-positive) deleted for the PWS-locus. (F) FISH on flow sorted mCherry-positive MAT-deletion line 5–9, almost all cells having the PWS-deletion. (G) INS-1 deletion lines 5–5 and 5–9 express PWS-imprinted genes. (H) Schematic of origin for MAT-deletion INS-1 lines 5–5 and [file pgen.1010710.s001.jpg]

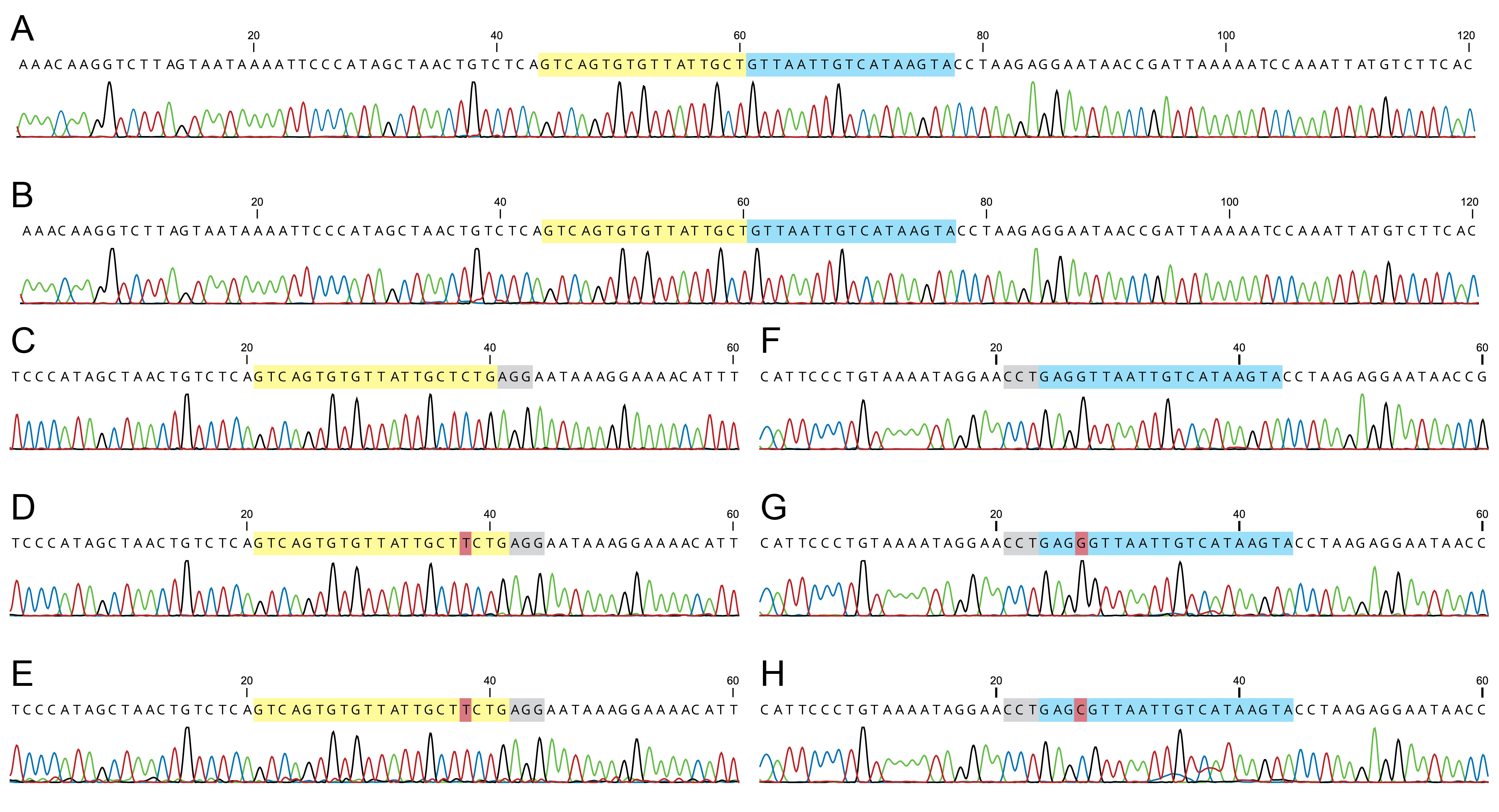

Supplement: S2 Fig — (A-H) Sanger sequence traces are shown highlighting sgRNA1 (yellow), sgRNA3 (blue), SpCas9 NGG PAMs (grey), and insertion mutations (pink). (A-B) Maternal-deletion allele of clonal lines 5–9 (A) and 5–5 (B) are identical canonical deletions with 3.16 Mb deleted between sgRNA1 and sgRNA3 due to a breakpoint from DNA repair of DSBs occurring at each position 3-nt upstream of the PAM (PAM-3) nuclease sites (see S1A Fig). Deletion-PCR breakpoint fragments are from the Fig 1C gel. (C-E) Sequence of intact sgRNA1 site in parental INS-1 (C), and scarred alleles with a single T/A insertion at the canonical PAM-3 DSB position (pink highlight) in clonal lines 5–9 (D) and 5–5 (E). Sequenced PCR fragments spanning sgRNA1 are from the gel in S1I Fig (F-H) Sequence of intact sgRNA3 site in parental INS-1 (F), and scarred alleles with a G/C insertion or a C/G insertion at the PAM-3 site (pink highlight) in line 5–9 (G) or line 5–5 (H), respectively. Sequenced PCR fragments spanning sgRNA3 are from the gel in S1I Fig It may be noted that as the deletions for 5–9 and 5–5 are on the maternal allele, the sgRNA1 and sgRNA3 scarred alleles can be inferred to occur on the paternal allele for each cell line. Further, as each of lines 5–5 and 5–9 have different sgRNA3 scarred alleles (despite sharing deletion breakpoints and sgRNA1 scarred allele mutations) then these two cell lines clearly arose as independent genome editing events. (JPG) [file pgen.1010710.s002.jpg]

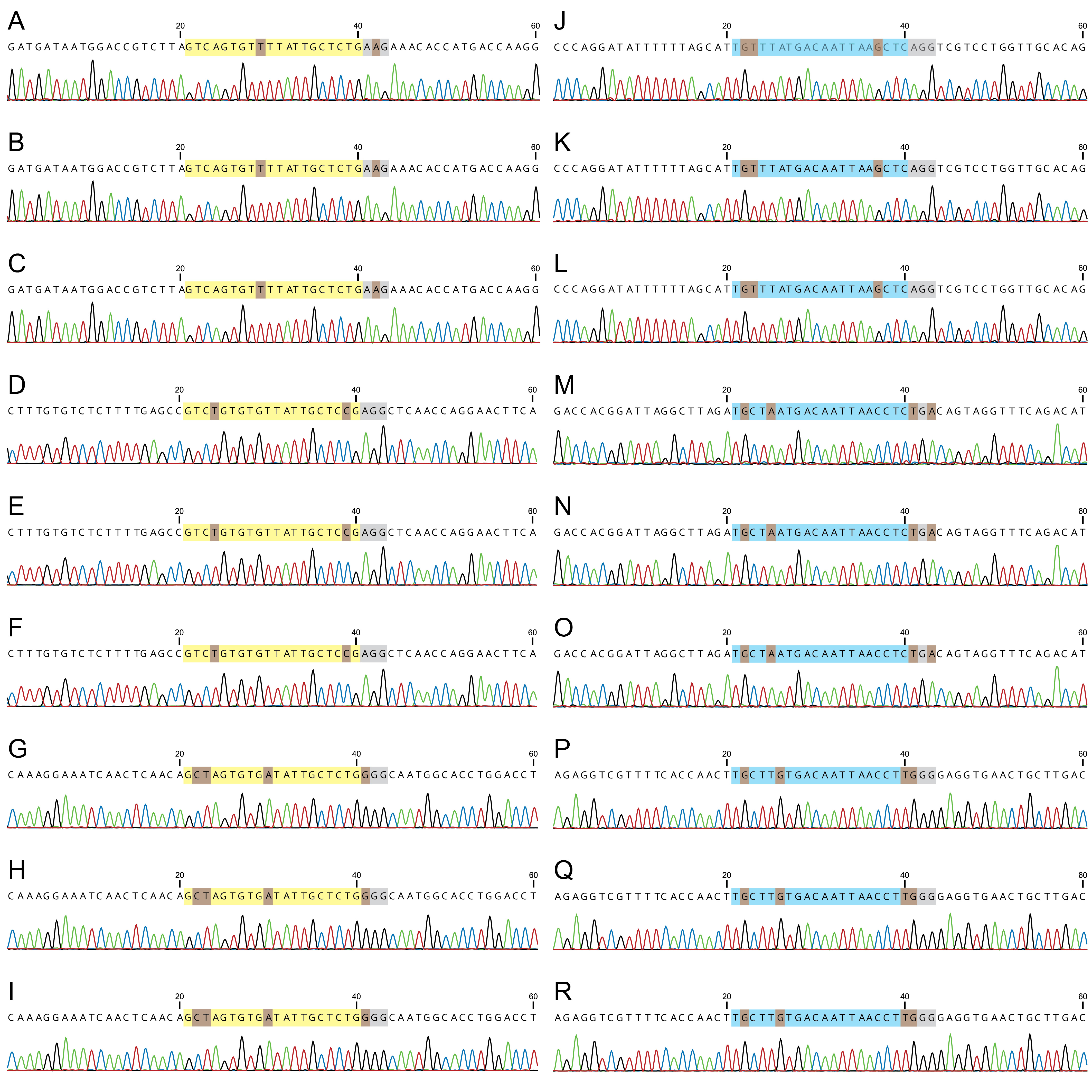

Supplement: S3 Fig — Sanger sequencing chromatographs of direct sequenced off-target genomic PCRs are shown for the top three ranked predicted off-target sites for sgRNA1 highlighted by the sgRNA seed (yellow) and for sgRNA3 (blue), with SpCas9 NGG PAMs (grey), and deviation in off-target from sgRNA sequence (brown). (A-C) Sequence of intact sgRNA1 off-target site at chromosome 2 position 115,451,401–115,451,423 (+; intergenic Gnb4-Actl6a) with 1 mismatch in parental INS-1 (A) and maternal (MAT)-deletion lines 5–9 (B), and 5–5 (C). (D-F) Sequence of intact sgRNA1 off-target site at chromosome 6 position 102,394,187–102,394,209 (-; intron Rgs6) with 2 mismatches in parental INS-1 (D) and MAT-deletion lines 5–9 (E), and 5–5 (F). (G-I) Sequence of intact sgRNA1 off-target site at chromosome 1 position 185,582,894–185,582,916 (+; intergenic Htra1-Dmbt1) with 3 mismatches in parental INS-1 (G) and MAT-deletion lines 5–9 (H), and 5–5 (I). (J-L) Sequence of intact sgRNA3 off-target site at chromosome 19 position 19,693,031–19,693,053 (+; intergenic Cbln1-N4bp1) with 3 mismatches in parental INS-1 (J) and MAT-deletion lines 5–9 (K), and 5–5 (L). (M-O) Sequence of intact sgRNA3 off-target site at chromosome 6 position 88,102,670–88,102,692 (+; intron Sos2) with 2 mismatches in parental INS-1 (M) and MAT-deletion lines 5–9 (N), and 5–5 (O). (P-R) Sequence of intact sgRNA3 off-target site at chromosome 9 position 105,005,251–105,005,273 (+; intron Tmem232) with 3 mismatches in parental INS-1 (P) and MAT-deletion lines 5–9 (Q), and 5–5 (R). In all off-target sites analyzed for both sgRNA1 and sgRNA3 there was no evidence of CRISPR-Cas9 induced dsDNA break repair resulting in small insertion-deletion events. (JPG) [file pgen.1010710.s003.jpg]

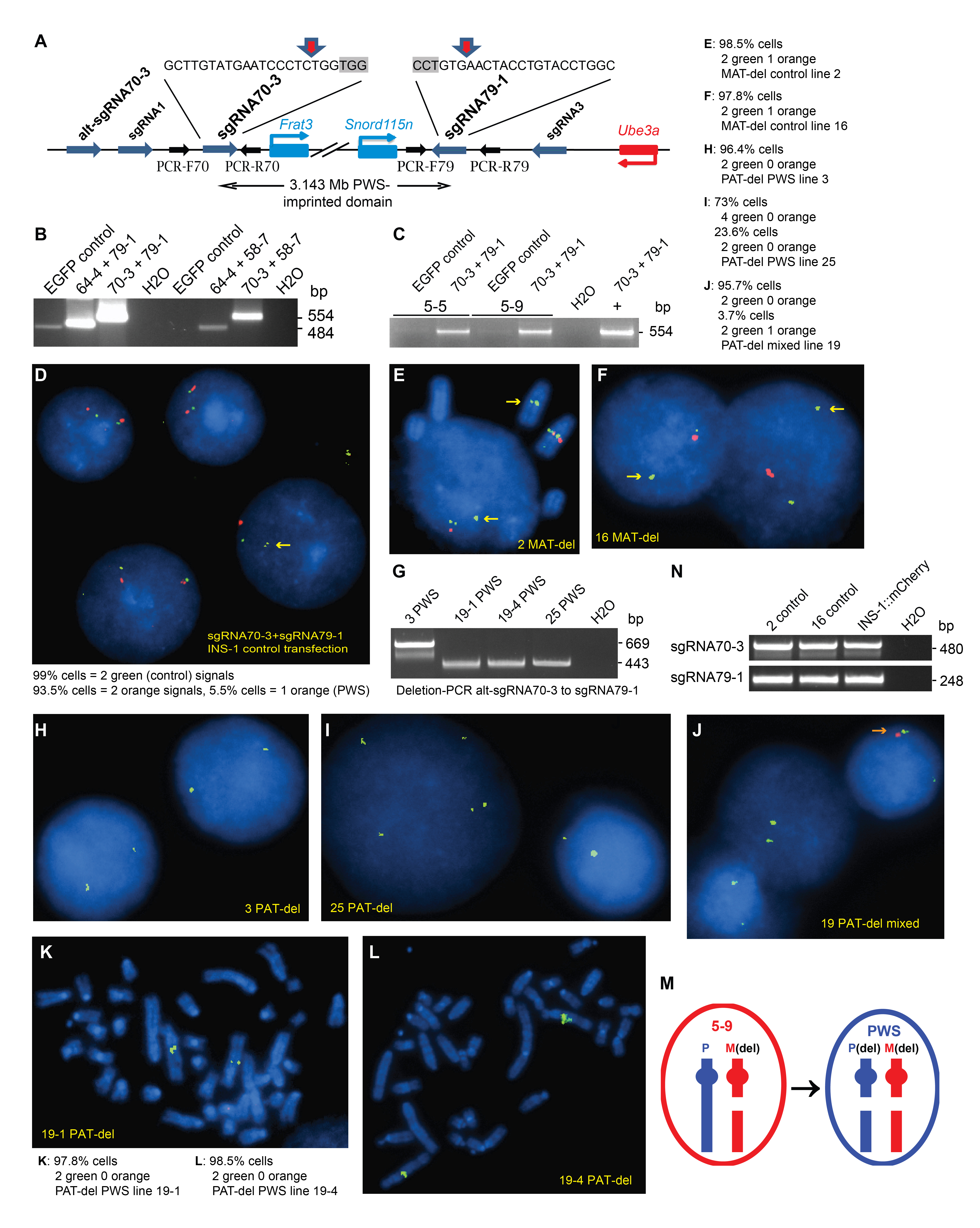

Supplement: S4 Fig — (A) Map of proximal sgRNA70-3 and distal sgRNA79-1 targeting sites on the paternal (PAT)-allele. Both sgRNA sites are absent on the maternal allele of lines 5–5 and 5–9 (S1 Fig), providing the PAT-allele specificity. Further proximal, the alt-sgRNA70-3 site differs at two 5′ nucleotides of the sgRNA and is part of a segmental-duplication (to be described elsewhere; manuscript in preparation). All symbols are as for S1A Fig (B) Deletion-PCR assays for a CRISPR/Cas9 screen of four pairs of sgRNAs flanking the 3.143 Mb PWS-domain, using control INS-1 cells. EGFP represents control transfections. Of the four pairs of sgRNAs, the highest efficiency is obtained for sgRNA70-3 + sgRNA79-1. (C) Deletion-PCR assay following transfection of MAT-del lines 5–5 and 5–9 using the CRISPR (sgRNA70-3 + sgRNA79-1)/Cas9-expressing vector shows specific targeting of the paternal allele of the PWS-domain. The positive (+) control is from S4B Fig (D-F, H-L) FISH studies using rat FISH probes, as for S1B–S1F Fig (D) FISH for parental INS-1 cells transfected with plasmid vectors expressing EGFP (not shown) or CRISPR (sgRNA70-3 + sgRNA79-1)/Cas9. Control cells for both probes (not shown) and the control probe (green) show diploid signals in virtually all cells, whereas for the PWS-probe (orange) in genome edited cells 5% of cells display an interphase with a PWS-region deletion (yellow arrow). (E-N) Studies on clonal lines derived from transfection of the MAT-del 5–9 cell line with an EGFP control vector (E-F, N) or with the CRISPR (sgRNA70-3 + sgRNA79-1)/Cas9-expressing vector (G-M). (E) Interphase and partial metaphase FISH for control line 2 with a MAT-deletion in virtually all cells. (F) FISH for control line 16 with a MAT-deletion in most cells. (G) Deletion-PCR for PAT-del line 3 using alt-sgRNA70-3 specific F and sgRNA79-1 R primers. As compared to the faint deletion breakpoint band using a sgRNA70-3 F PCR primer (Fig 1E), this alternate (alt) assay provides a high degree of specifici [file pgen.1010710.s004.jpg]

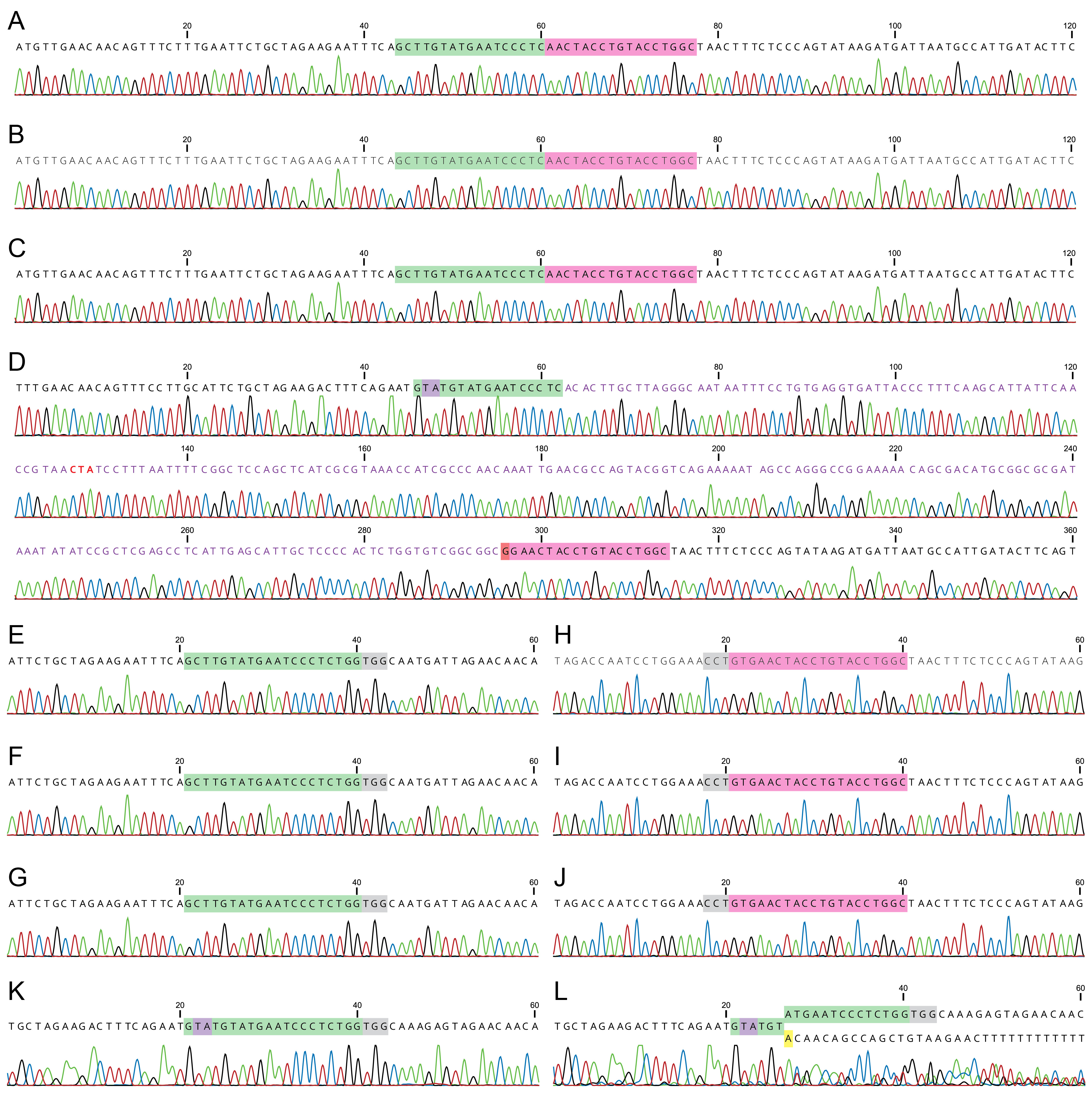

Supplement: S5 Fig — (A-J) Sanger sequence traces are shown highlighting sgRNA70-3 (green), sgRNA79-1 (pink), and SpCas9 NGG PAMs (grey). (A-C) Paternal (PAT)-del allele of clonal line 19–1 (A), 19–4 (B), and 25 (C) are identical canonical deletions with 3.143 Mb deleted between the sgRNA70-3 and sgRNA79-1 PAM-3 nuclease sites. Deletion-PCR breakpoint fragments are from gels such as shown in Fig 1E. (D) PAT-del line 3 generated an unexpected proximal deletion breakpoint at the canonical PAM-3 position of an alternate (alt) sgRNA70-3 site upstream of Frat3, that maps ~ 125 kb upstream of the sgRNA70-3 position (see Figs 1A and S4A). The alt-sgRNA70-3 site differs only at 2 of the most 5′ nucleotides of the sgRNA (purple highlight) and is part of an uncharacterized segmental-duplication. The line 3 genome-editing event with a 3.268 Mb deletion has at the breakpoint a 233- or 234-bp insertion of sequence with 97% identity to E. coli (purple sequence, with gsiD amber stop codon indicated in red), followed by a distal deletion breakpoint with a single G/C nucleotide insertion or polymorphism in E. coli sequence (orange highlight) that occurred at a DSB at the PAM-2 position of the distal sgRNA79-1 site (pink highlight). The deletion-PCR breakpoint fragment for the PAT-del line 3 is from the gel in S4G Fig (E-G) Sequence of intact sgRNA70-3 site in parental INS-1 (E) and maternal (MAT)-deletion (del) control lines 2 (F) and 16 (G). Sequenced PCR fragments spanning sgRNA70-3 are from the gel in S4N Fig (H-J) Sequence of intact sgRNA79-1 site in parental INS-1 (H) and MAT-del control lines 2 (I) and 16 (J). Sequenced PCR fragments spanning sgRNA79-1 are from the gel in S4N Fig (K-L) Sanger sequence traces of the alt-sgRNA70-3 site by direct Sanger sequencing of a genomic PCR product showing an intact site in parental INS-1 (K), and a hemizygous scarred allele with a 28-nt deletion (leaving 6 nt intact at the 5’-end of the alt-sgRNA70-3 sgRNA site) in clonal PAT-del line 19–1 (L). Sequence from [file pgen.1010710.s005.jpg]

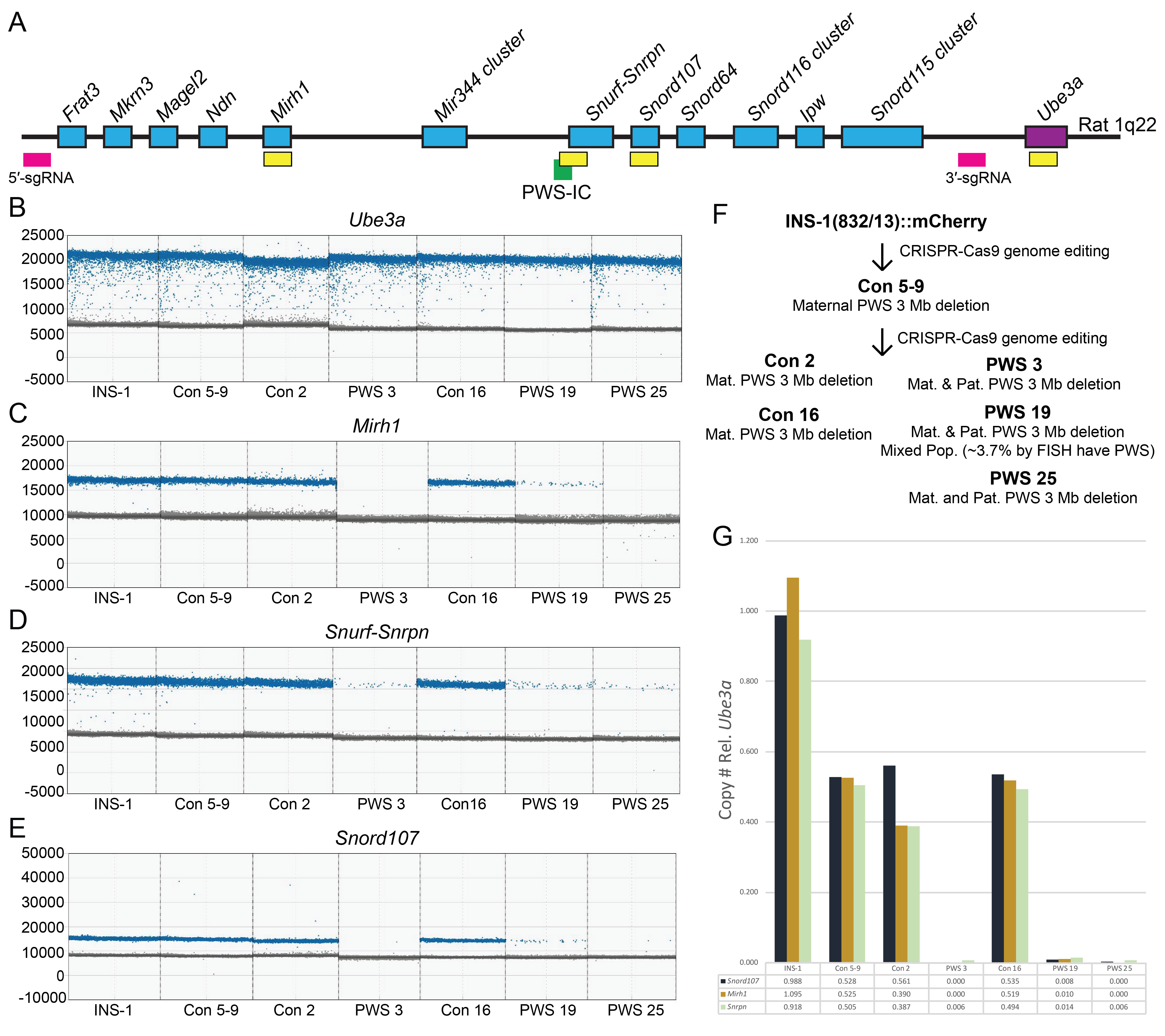

Supplement: S6 Fig — (A) Schematic of the PWS-imprinted domain with paternally expressed genes in blue and the maternally expressed Ube3a in purple. The positions of the sgRNAs that mark the PWS-deletion breakpoints are indicated by pink boxes, the PWS-IC by a green box, and the four loci examined by ddPCR by yellow boxes. (B) Genomic ddPCR 1d amplitude plot for Ube3a, localized outside of the distal PWS-deletion breakpoint and hence intact in all INS-1 cell lines. (C-E) Genomic ddPCR 1d amplitude plots for (C) Mirh1, (D) Snurf-Snrpn, and (E) Snord107, each localized within the PWS-deletion region. (F) Schematic showing generation in an initial CRISPR/Cas9 genome editing screen of clonal control (Con) cell line 5–9 with an ~ 3 Mb deletion of the PWS-domain on the maternal allele, and additional clonal cell lines generated from a second CRISPR/Cas9 genome editing screen. The latter include two further control lines, 2 and 16, each with a PWS-domain deletion on the maternal allele, and three independent homozygous deletion sublines 3, 19 and 25. (G) Graph of genomic ddPCR for the PWS-region demonstrating that sublines 3 and 25 are pure populations of cells with a homozygous PWS-region deletion. However, whereas most cells in subline 19 have a homozygous PWS-deletion, there is a small percentage of cells having an intact PWS-region (i.e., derived from the 5–9 parental line) as a mixed population. FISH also confirmed 3.7% of cells with an intact PWS-region in subline 19 (see S4J Fig). Note that subline 19 then had a further screen by plating single cells in a 96-well plate and selecting clonal lines with a homozygous PWS-deletion (i.e., 19–1, 19–2, 19–3, 19–4, 19–5). (JPG) [file pgen.1010710.s006.jpg]

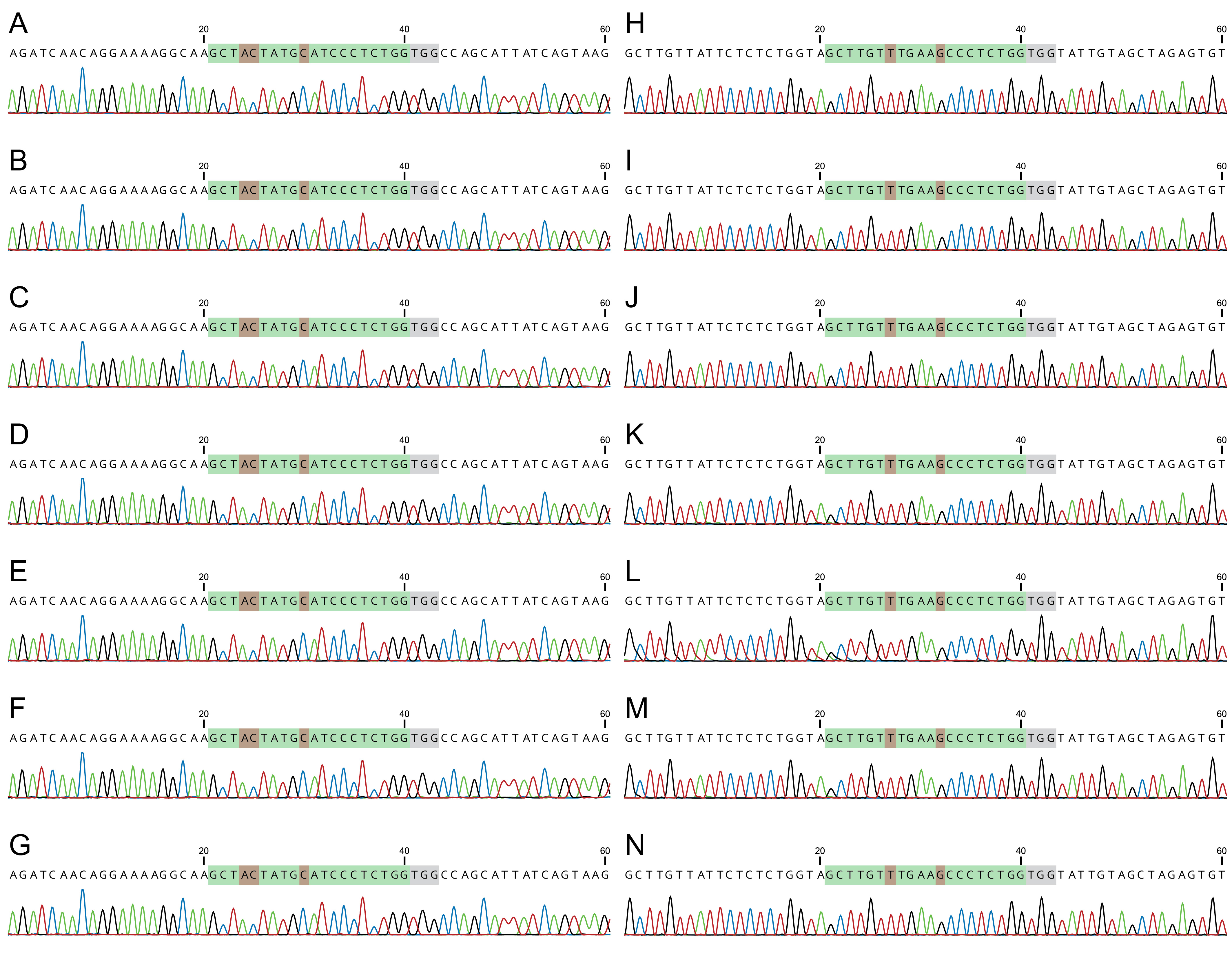

Supplement: S7 Fig — (A-N) Sanger sequence traces are shown for the top 2 ranked predicted off-target sites for sgRNA70-3 highlighted by the sgRNA seed (green), SpCas9 NGG PAMs (grey), and deviation in off-target from sequence from sgRNA70-3 (brown). (A-G) Sequence of intact sgRNA70-3 off-target site at chromosome 1 position 178,678,292–178,678,314 (-; intron Hs3st4) with 3 mismatches in parental INS-1 (A) and maternal (MAT)-deletion lines 5–9 (B), line 2 (C), line 16 (D), and paternal (PAT)-deletion lines 3 (E), 19–1 (F) and 19–4 (G). (H-N) Sequence of intact sgRNA70-3 off-target site at chromosome 8 position 72,506,613–72,506,635 (+; intron Tcf12) with 2 mismatches in parental INS-1 (H) and MAT-deletion lines 5–9 (I), line 2 (J), line 16 (K), and PAT-deletion lines 3 (L), 19–1 (M) and 19–4 (N). (JPG) [file pgen.1010710.s007.jpg]

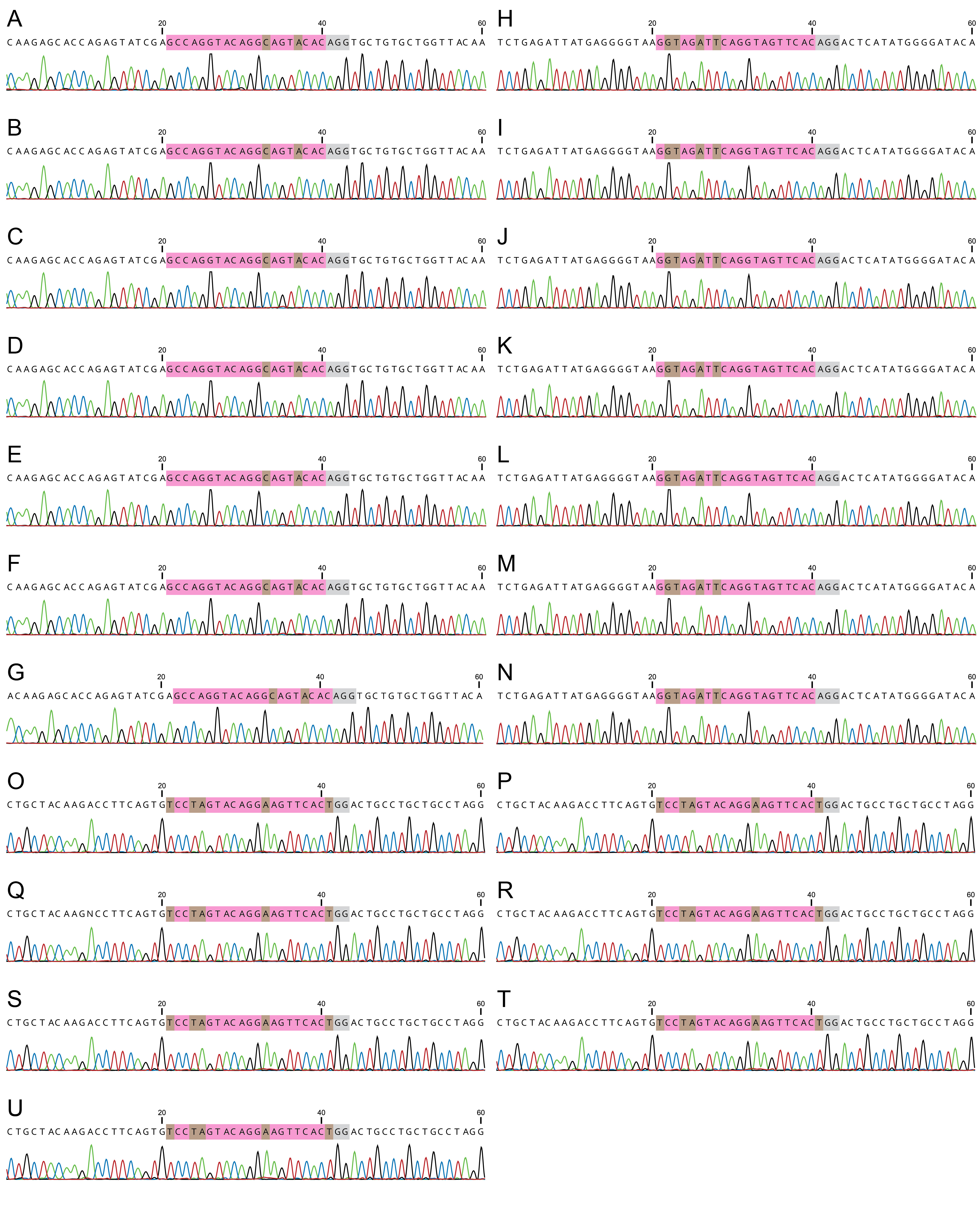

Supplement: S8 Fig — (A-U) Sanger sequence traces are shown for the top 3 ranked predicted off-target sites for sgRNA79-1 highlighted by the sgRNA seed (pink), SpCas9 NGG PAMs (grey), and deviation in off-target from sequence from sgRNA79-1 (brown). (A-G) Sequence of intact sgRNA79-1 off-target site at chromosome 9 position 58,847,915–58,847,937 (+; intergenic Satb2-RGD1306941) with 2 mismatches in parental INS-1 (A) and maternal (MAT)-deletion lines 5–9 (B), line 2 (C), line 16 (D), and paternal (PAT)-deletion lines 3 (E), 19–1 (F) and 19–4 (G). (H-N) Sequence of intact sgRNA79-1 off-target site at chromosome 3 position 128,425,398–128,425,420 (+; intron Macrod2) with 4 mismatches in parental INS-1 (H) and MAT-deletion lines 5–9 (I), line 2 (J), line 16 (K), and PAT-deletion lines 3 (L), 19–1 (M) and 19–4 (N). (O-U) Sequence of intact sgRNA79-1 off-target site at chromosome 2 position 47,166,018–47,166,040 (+; intergenic Itga1-Isl1) with 4 mismatches in parental INS-1 (O) and MAT-deletion lines 5–9 (P), line 2 (Q), line 16 (R), and PAT-deletion lines 3 (S), 19–1 (T) and 19–4 (U). (JPG) [file pgen.1010710.s008.jpg]

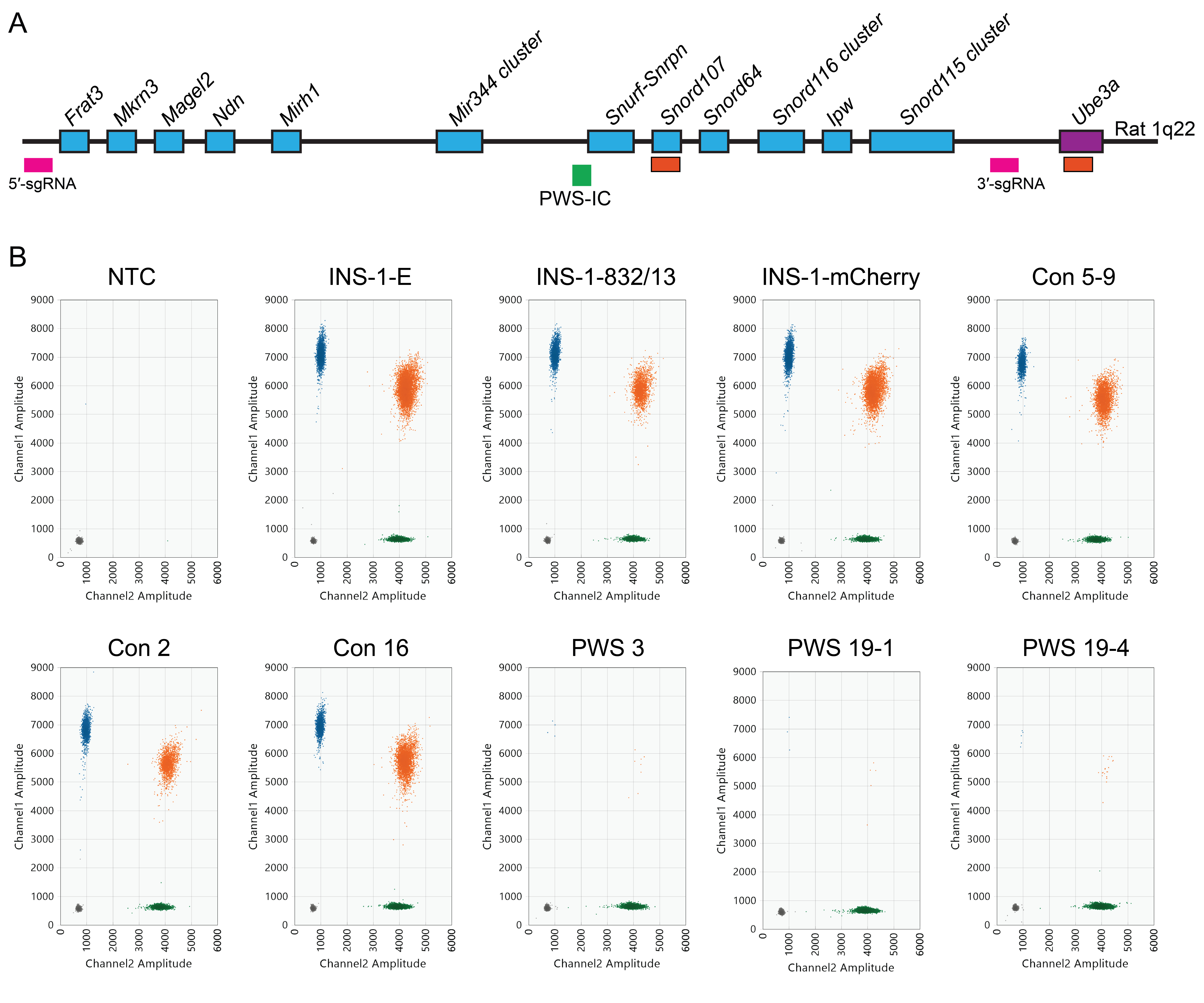

Supplement: S9 Fig — (A) Schematic of the PWS-imprinted domain with paternally expressed genes in blue and the maternally expressed Ube3a in purple. The positions of the sgRNAs that mark the PWS-deletion breakpoints are indicated by pink boxes, the PWS-IC by a green box, and the probes used for two loci that were examined by TaqMan ddPCR by orange boxes. (B) ddPCR 2d plots for TaqMan probe copy number assay with absorbance amplitude for channel 1 (Snord107, FAM) on the y-axis and channel 2 (Ube3a, HEX) on the x-axis. Blue dots denote Snord107 positive droplets, green dots represent Ube3a positive droplets and orange indicate double positive droplets. Note the absence of channel 1 Snord107 positive (blue or orange droplets) in the PWS lines, and 50% reduction of channel 1 positive droplets in maternal-deletion control (Con) lines (also see Fig 1F for graphical data). NTC, no template control. (JPG) [file pgen.1010710.s009.jpg]

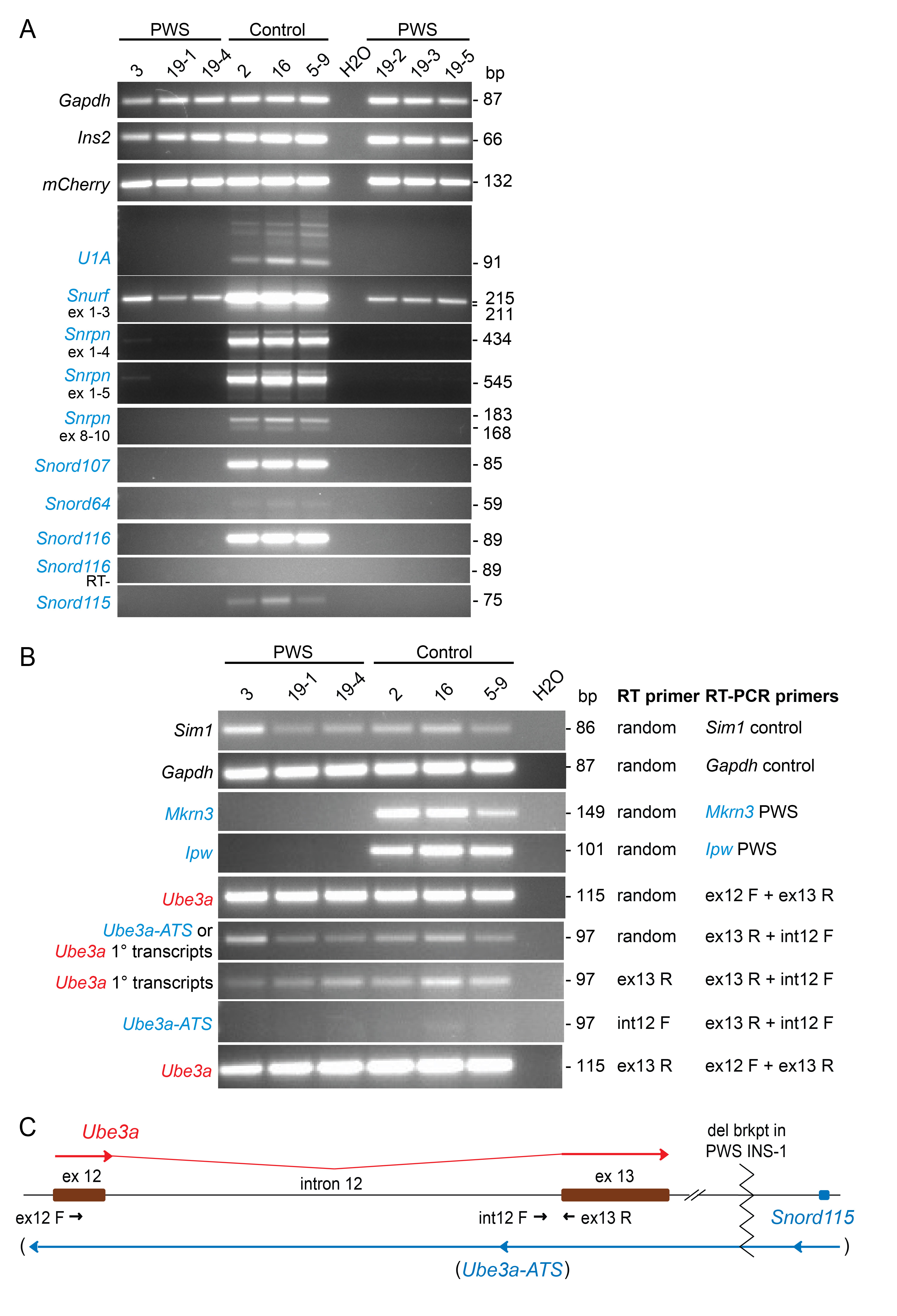

Supplement: S10 Fig — (A) RT-PCR analyses of 7 PWS-imprinted genes (U1A, Snurf, Snrpn, Snord107, Snord64, Snord116, Snord115; see map in Fig 1A), rat Ins2, mCherry transgene, and Gapdh control gene in the expanded INS-1 panel of 9 cell lines. U1 represents an alternate upstream (U) promoter-first exon that splices into Snurf-Snrpn exon 2 and has multiple duplicate U1 copies in rodents. Abbreviations: ex, exon; RT-, PCR control using RNA not treated with reverse-transcriptase. The absence of bands in the RT- assay for the multicopy, tandemly repeated Snord116 locus rules out genomic DNA contamination in the RNA. Note that the RT-PCR gel results for 4 genes (Gapdh, Ins2, Snrpn ex 8–10, Snord116) in the 6-cell line panel used in this entire study are reproduced from Fig 1G for direct comparison to the remaining genes in the PWS domain that are presented here, plus this dataset has 3 additional clonal PWS INS-1 cell lines (19–2, 19–3, 19–5). Similarly, while the Snurf ex 1–3 results are shown here for the expanded panel of nine INS-1 cell lines, these same results for the 6-cell line panel used in this entire study are reproduced in S11B Fig for comparison to other loci examined in that dataset. (B) RT-PCR analyses of 2 PWS-imprinted genes (Mkrn3, Ipw) as well as Ube3a and Ube3a-ATS loci (see maps in Figs 1A and S10C), and control genes (Gapdh, Sim1) in the 6-cell line panel used in this entire study. The upper 6 rows of gels are from standard RT-PCR assays that use random hexamer primers for the RT primer and typical gene-specific RT-PCR primers, while the lower 3 rows of gels are from RT-PCR assays that use a strand-specific primer for RT followed by RT-PCR with either a Ube3a (ex12 F + ex13 R) or Ube3a-ATS (ex13 R + int12 F) primer set. Combined, the data show that transcripts from the Ube3a-ATS-region that are detected when using random primers for RT derive from Ube3a primary (1°) transcripts since there is no expression from the Ube3a-ATS strand. (C) Map of the Ube3a and Ube3a-ATS locu [file pgen.1010710.s010.jpg]

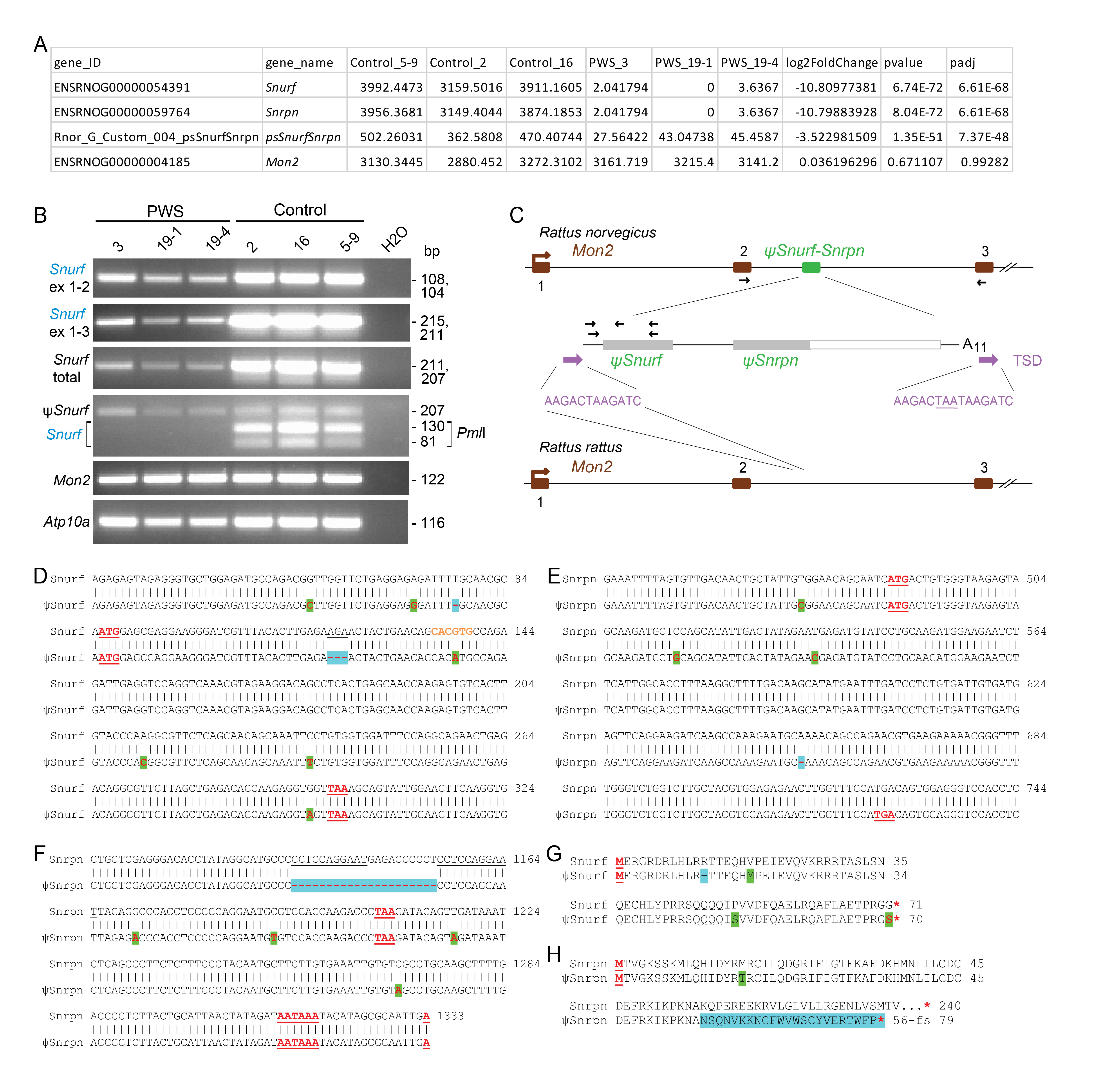

Supplement: S11 Fig — (A) RNA-seq analysis using a custom rat genome build identifies a ψSnurf-Snrpn locus expressed in PWS and control INS-1 lines. In contrast, the imprinted Snurf and Snrpn loci are only expressed in the control INS-1 lines. Expression for the pseudogene host gene, Mon2, is also shown. (B) RT-PCR with gel analysis for 3 amplicons from Snurf as well as ψSnurf-ψSnrpn, Mon2 and Atp10a in the INS-1 panel. The first 2 rows show primer sets designed to amplify Snurf exons (ex) 1–2 and 1–3, while row 3 shows RT-PCR with a primer set designed to amplify both Snurf and ψSnurf sequences; the latter RT-PCR products were then digested with the PmlI restriction endonuclease which distinguishes between Snurf and ψSnurf products. Note that Atp10a is significantly reduced in PWS vs. control lines (also see S1 and S2 Tables). (C) Map location of the expressed ψSnurf-ψSnrpn locus (green box) within the brown rat (Rattus norvegicus) Mon2 gene (brown boxes are 5’ exons 1–3). In contrast, the black rat (Rattus rattus) has no pseudogene insertion and a single copy of the Target Site Duplication (TSD, purple arrows) present in the brown rat genome at the 5’-end of the inserted pseudogene. Black arrows represent PCR primers, while the underlined TAA nucleotides represents a tandem duplication in the 3’-TSD copy. (D) DNA sequence of ψSnurf transcripts in PWS INS-1 lines compared to the endogenous Snurf gene. Green shading of red nucleotides indicates missense mutations, and blue shading with a hyphen represents a nucleotide deletion. Orange font represents the PmlI cleavage site specifically in the Snurf cDNA sequence. Snurf start and stop codons are indicated. (E) DNA sequence identifies a frameshift from a single nucleotide deletion and premature stop codon in the 5’ ψSnrpn portion of the pseudogene. Symbols as for (D). (F) DNA sequence identifies a 21-nucleotide deletion in the 3’ ψSnrpn portion of the pseudogene. Symbols as for (D). (G) Potential ψSnurf amino acid sequence encoded by the ψ [file pgen.1010710.s011.jpg]

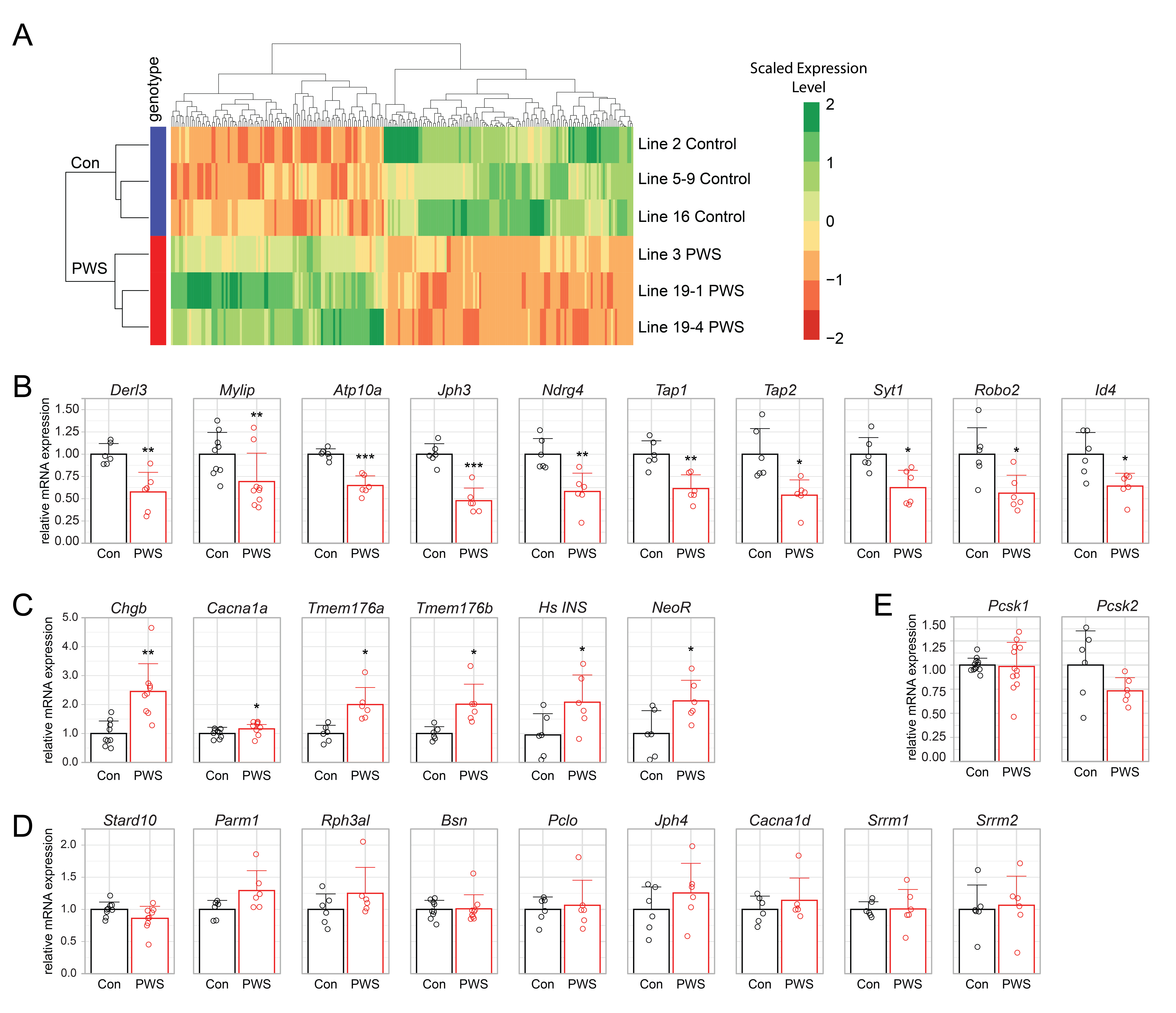

Supplement: S12 Fig — (A) Heatmap clustergram of 228 DEGs demonstrates tight clustering of PWS vs. control groups (Padj < 0.05). Scale: green (enriched) to red (depleted). RNA-seq was performed for 3 PWS (3, 19–1, 19–4) vs. 3 control (5–9, 2, 16) INS-1 cell lines. (B-E) Quantitative gene expression analyses for control (Con, black; 5–9, 2, 16) vs. PWS (red; 3, 19–1, 19–4) determined by RT-ddPCR normalized to Gpi levels and to the average expression in control INS-1 lines, for (B-E) panels of additional candidate DEGs. The latter include validation of (B) 10 down-regulated genes (Derl3, Mylip, Atp10a, Jph3, Ndrg4, Tap1, Tap2, Syt1, Robo2, Id4), as well as (C) for a set of 4 upregulated DEGs involved in the secretory pathway (Chgb, Cacna1a, Tmem176a, Tmem176b), with apparent upregulation for an exogenous human insulin (Hs, Homo sapiens)-neomycin resistance (NeoR) transgene in the INS-1 lines, although the latter is artifactual due to transgene silencing in line 16 (see S16A,S16I–S16K and S16M Fig). (D,E) However, the RT-ddPCR analyses failed to validate (D) several candidate upregulated DEGs from the RNA-seq data (Fig 4A), or (E) candidates for downregulated DEGs based on data from PWS iPSC-derived neuronal and Snord116-deficient mouse models [60,129]; likewise, another mouse study [130] did not confirm Pcsk1. Statistical comparison by Welch’s t-test: *, P < 0.05; **, P < 0.005; ***, P < 0.0005. (JPG) [file pgen.1010710.s012.jpg]

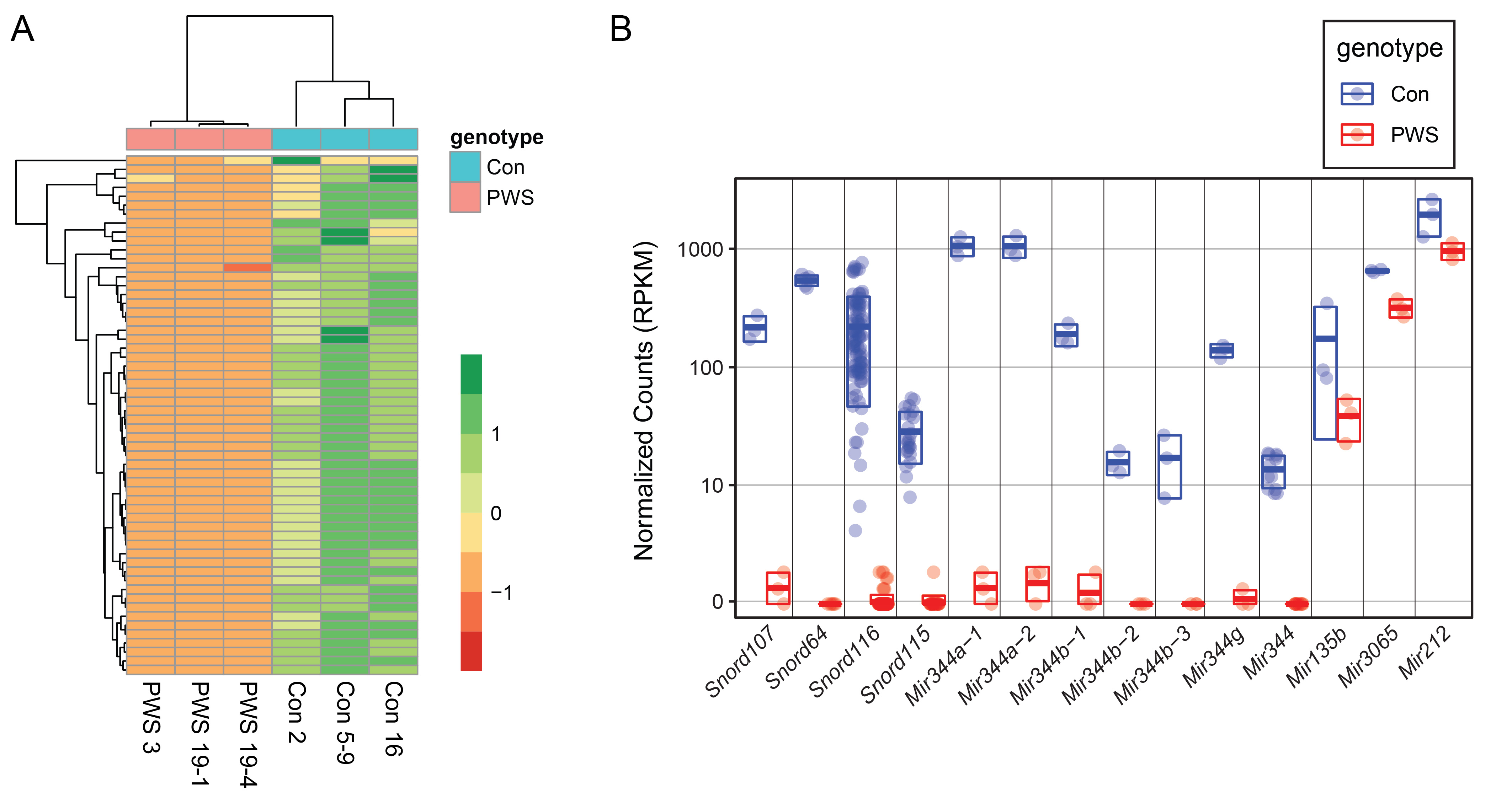

Supplement: S13 Fig — (A) Heatmap clustergram of 58 differentially expressed miRNAs and snoRNAs demonstrates tight clustering of PWS vs. control groups (Padj < 0.1). Scale: green (enriched) to red (depleted). Small RNA-seq was performed for 3 PWS (3, 19–1, 19–4) vs. 3 control (5–9, 2, 16) INS-1 cell lines. (B) Normalized expression counts for the top 14 significant differentially expressed miRNAs and snoRNAs, of which only 3 miRNAs identified are not encoded within the PWS-imprinted domain. Box charts of control (blue) and PWS (red) genotypes are shown with underlying data points for each sample. For the multicopy Snord115, Snord116, and Mir344 genes the data for the paralogs were binned. (JPG) [file pgen.1010710.s013.jpg]

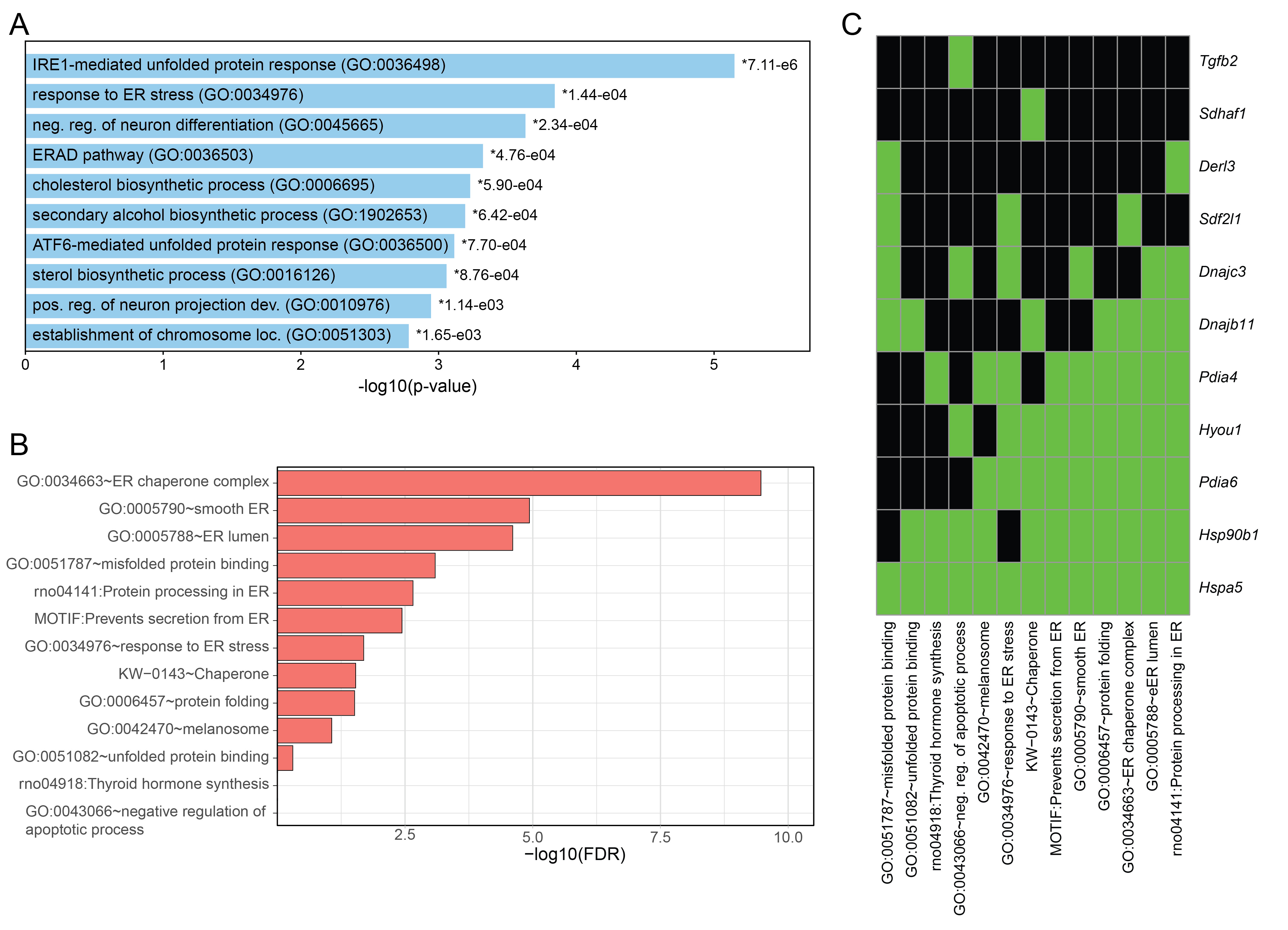

Supplement: S14 Fig — (A) EnrichR gene ontology (GO Biological Process 2018 version) terms enriched in down-regulated DEGs highlighting ER stress mediators including IRE1, ATF6, and ER-associated protein degradation (ERAD) pathways. (B) DAVID analysis reveals a functional annotation cluster (Enrichment Score: 4.376) and (C) considerable overlap of ER functions and unfolded protein response components among the functional annotation groups (as represented by the green boxes). Gene ontology analysis performed on a gene set of 81 downregulated DEGs filtered by adjusted P value < 0.1 and Fold Change < 0.75 (log2FC < -0.415) with all PWS genes removed. (JPG) [file pgen.1010710.s014.jpg]

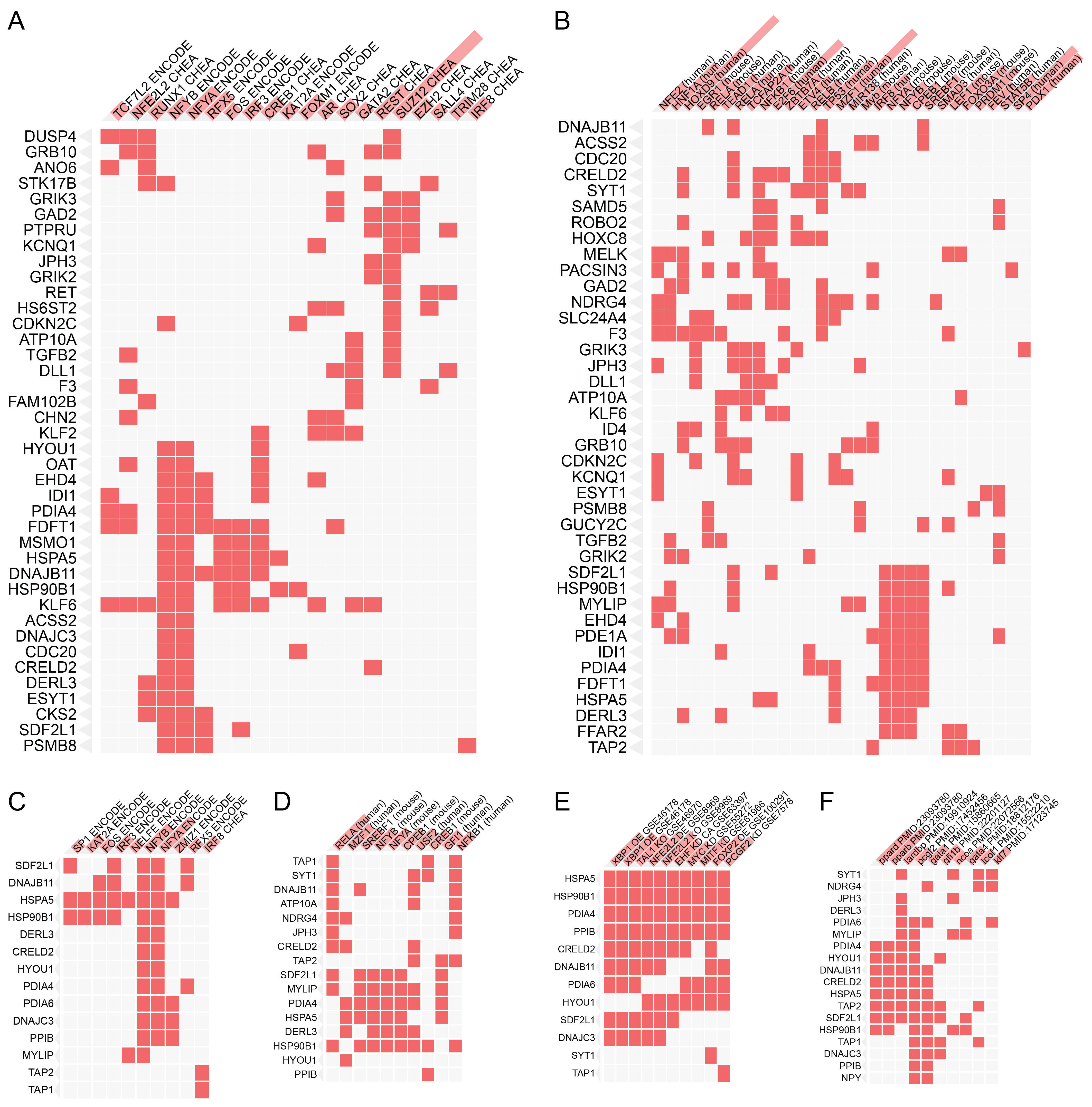

Supplement: S15 Fig — (A-B) Enrichr transcription factor (TF) analysis of 94 down-regulated non-PWS DEGs (Padj < 0.10 and an absolute fold-change (FC) >1.25) shown as clustergrams of (A) top 20 ENCODE and ChEA Consensus TFs from ChIP-X or (B) top 30 TRANSFAC and JASPAR PWMs enriched in the gene set. (C-F) Enrichr TF analysis for 20 RT-ddPCR validated down-regulated genes (from Figs 4D, 4E and S12B: Hspa5, Hsp90b1, Pdia4, Pdia6, Ppib, Creld2, Sdf2l1, Dnajb11, Dnajc3, Hyou1, Npy, Iapp, Derl3, Mylip, Atp10a, Jph3, Ndrg4, Tap1, Tap2, Syt1. Further analysis of validated DEGs enriched for TF binding sites from (C) ENCODE and ChEA Consensus TFs, (D) TRANSFAC and JASPAR motifs, (E) TF Perturbations Followed by Expression (DE, differential expression; KD, knockdown; KO, knockout; OE, over-expression) with GEO accession listed, and (F) TF-LOF Expression from GEO with PMID reference appended (LOF, loss of function). Clustergrams generated based on enrichment P-value significance which shows as the red highlight of TF names. Notably in the downregulated genes in PWS INS-1 cells a prominent cluster of genes including many ER chaperones were enriched for ATF6-cofactor NFYA and NFYB binding sites and other potential regulators including CPEB1, RFX5, IRF3, CREB1 and SREBF1 (see S15A–S15D Fig). Similarities to other model systems revealed analogous gene-expression changes including for Xbp1 perturbations in an adipose cell line [131] and the Pparb/d KO mouse pancreas [93] (see S15E–S15F Fig). (JPG) [file pgen.1010710.s015.jpg]

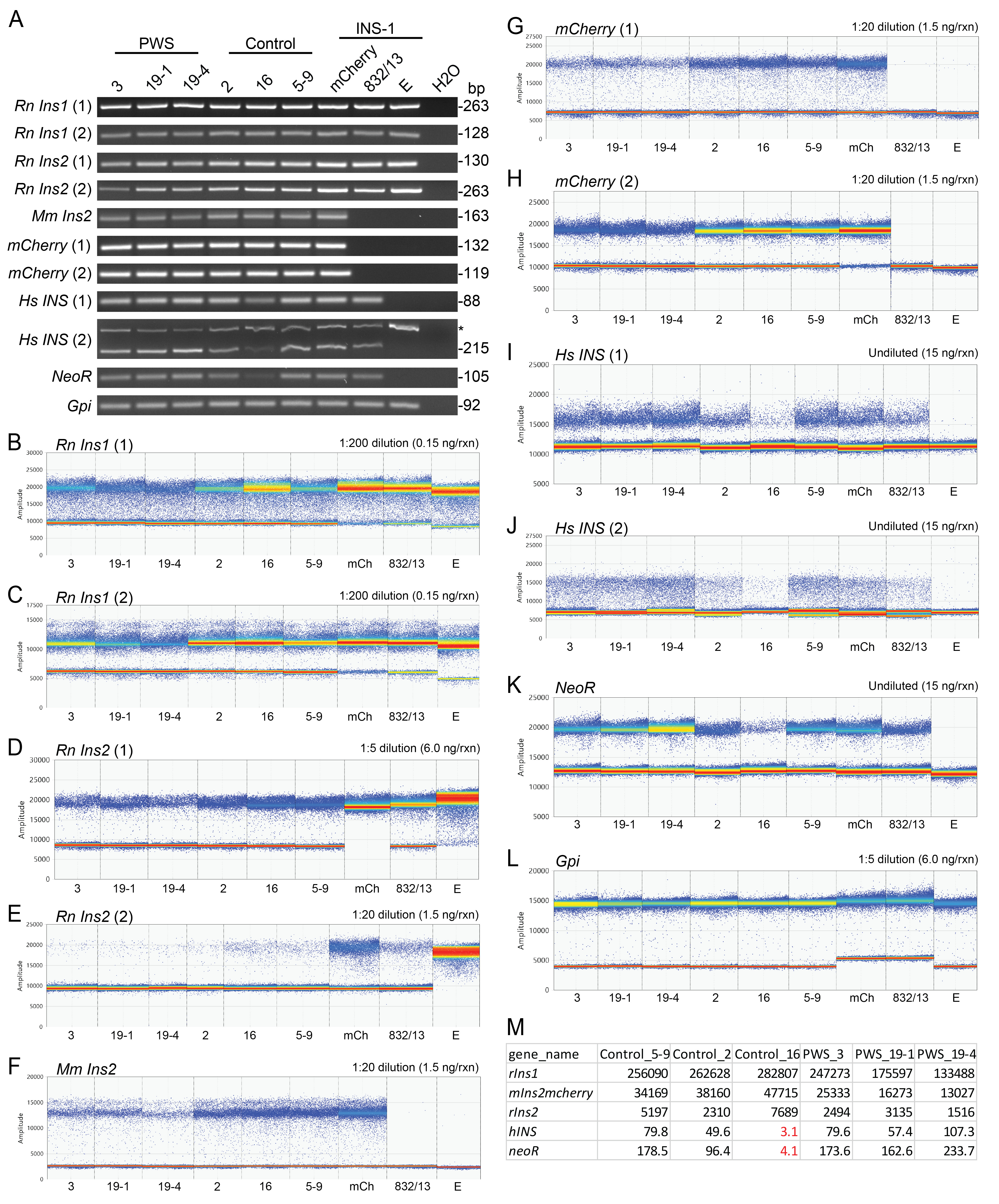

Supplement: S16 Fig — (A) RT-PCR with gel analysis for insulin genes. Abbreviations are: (1), (2), represent amplicons 1 and 2 for a given gene; mCherry, INS-1(832/13)::mCherry cell line; 832/13, INS-1(832/13) cell line; E, INS1-E cell line; Hs, Homo sapiens; Mm, Mus musculus; Rn, Rattus norvegicus; *, non-specific band in the indicated RT-PCR assay (note that this amplified product is not present using the same PCR primer pair in the RT-ddPCR assay shown in S16J Fig, likely due to different chemistry or annealing temperature in the two assays). Note that control line 16 has greatly reduced expression of the Hs INS (amplicons 1 and 2) and NeoR segments of the INS-NeoR transgene, as also seen in the RT-ddPCR data [see S16–S16K Fig] and RNA-seq data [see S16M Fig], likely reflecting epigenetic inactivation of the INS-NeoR transgene in a majority of cells for line 16 (as DNA analysis indicated the transgene remained present). (B-L) RT-ddPCR assays for the listed amplicons, with ddPCR performed using EvaGreen. All abbreviations are as for S16A. (B) RT-ddPCR assay for Rn Ins1 amplicon 1. (C) RT-ddPCR assay for Rn Ins1 amplicon 2. (D) RT-ddPCR assay for Rn Ins2 amplicon 1. (E) RT-ddPCR assay for Rn Ins2 amplicon 2. (F) RT-ddPCR assay for Mm Ins2. (G) RT-ddPCR assay for mCherry amplicon 1. (H) RT-ddPCR assay for mCherry amplicon 2. (I) RT-ddPCR assay for Hs INS amplicon 1. (J) RT-ddPCR assay for Hs INS amplicon 2. (K) RT-ddPCR assay for NeoR. (L) RT-ddPCR assay for control gene Gpi. (M) RNA-seq analysis of insulin genes expressed in the INS-1 lines. In addition to the endogenous rat (r) Ins1 and Ins2 genes, a custom rat genome build identified mouse (m) Ins2-mCherry and Hs INS-NeoR transgene mRNA levels. Red numbers indicate that control line 16 is an outlier with drastically reduced expression of the Hs INS-NeoR transgene (see S16A Fig legend). (JPG) [file pgen.1010710.s016.jpg]

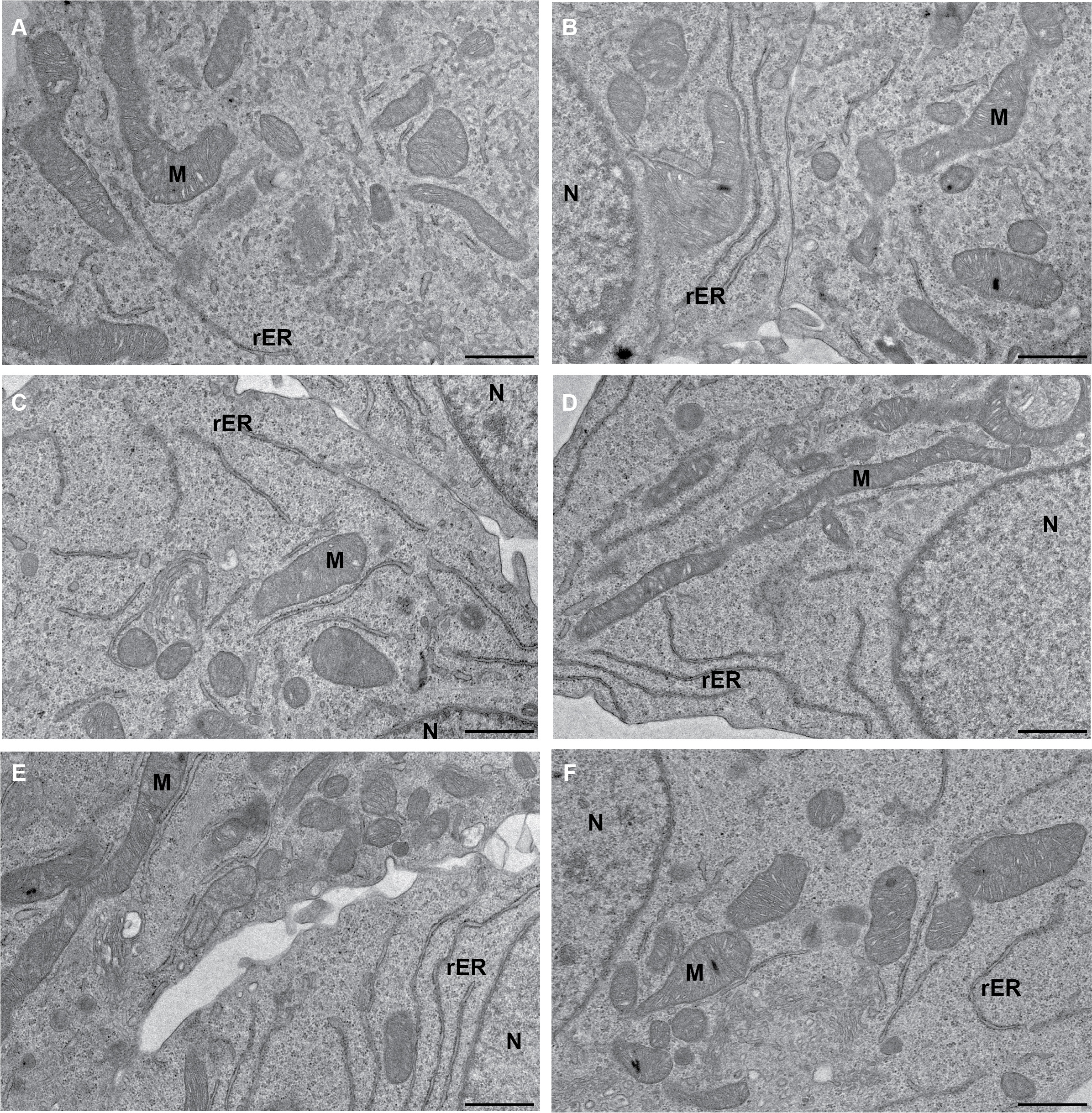

Supplement: S17 Fig — (A) Control line 5–9, (B) Control line 2, (C) Control line 16, (D) PWS line 3, (E) PWS line 19–1, (F) PWS line 19–4. The scale bar in the bottom right corner of each image is 800 nm, while abbreviations for features are: N, nucleus; M, mitochondria; rER, rough endoplasmic reticulum (for M and rER, only one label per image is shown). Key subcellular organelles of normal appearance include mitochondria and rough ER. These INS-1 832/13-derived cell lines, while producing and secreting significant insulin [34] (Figs 2–5) and other secretory peptides such as IAPP (Fig 3C and 3D) do not demonstrate clearly visible insulin secretory vesicles, likely because of the relative rarity of these compared to a number in the tens of vesicles detected in the INS-1E cell line [132] that produces much more abundant insulin (see Fig 5A). (JPG) [file pgen.1010710.s017.jpg]

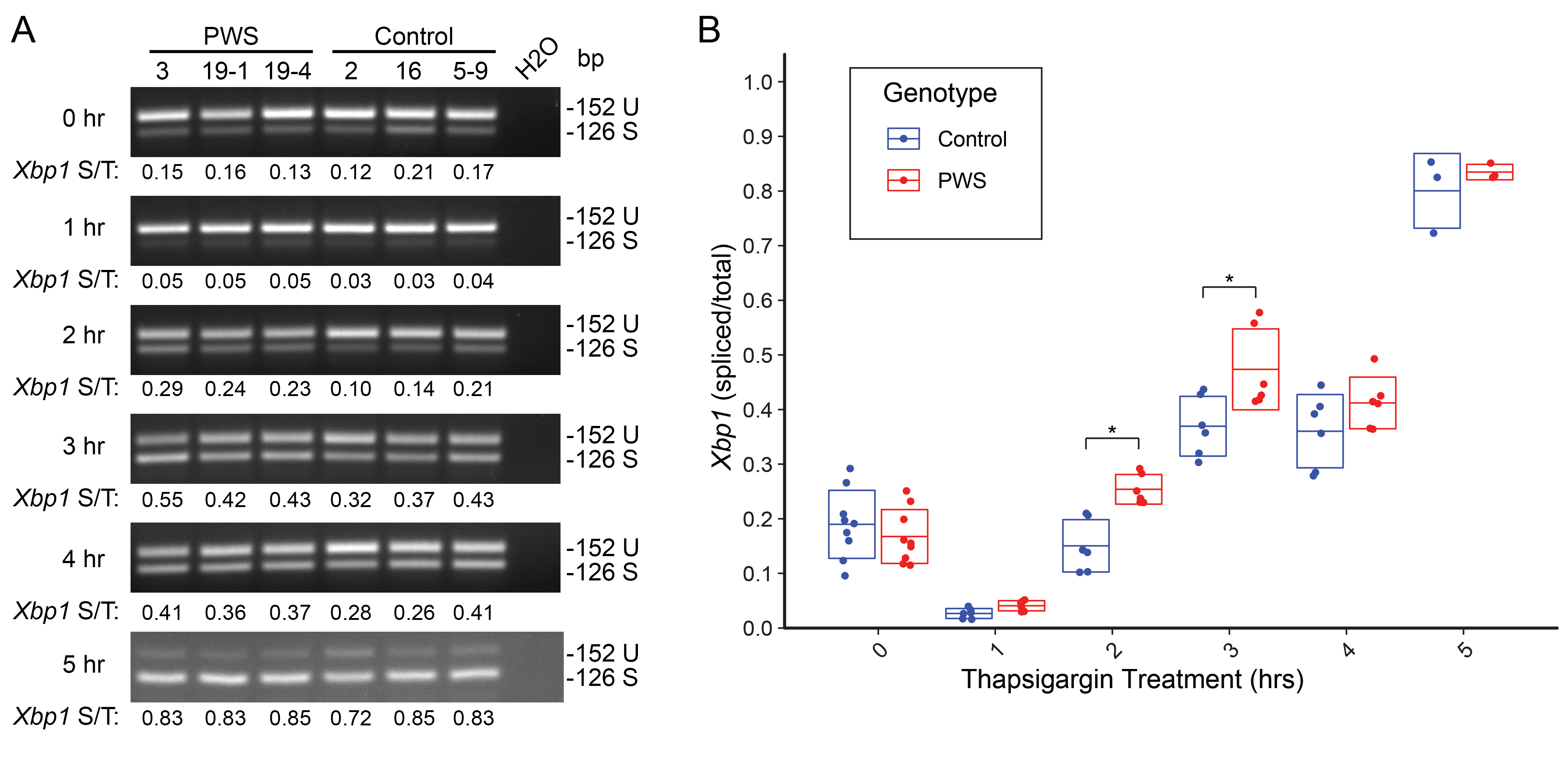

Supplement: S18 Fig — (A) Reverse transcription-PCR gel electrophoresis analysis of Xbp1 mRNA processing for exclusion of 26-nt of exon 4 from time 0 to 5 hours of thapsigargin treatment. Abbreviations: S, spliced; T, total; U, unspliced. (B) Time-course of Xbp1 mRNA activation as the ratio of spliced/total mRNA detected by RT-PCR in (A). Initially, at 1 hr of thapsigargin treatment Xbp1 mRNA levels fall due to mRNA turnover for both control and PWS cell lines. *, P < 0.05 as calculated by ANOVA. (JPG) [file pgen.1010710.s018.jpg]

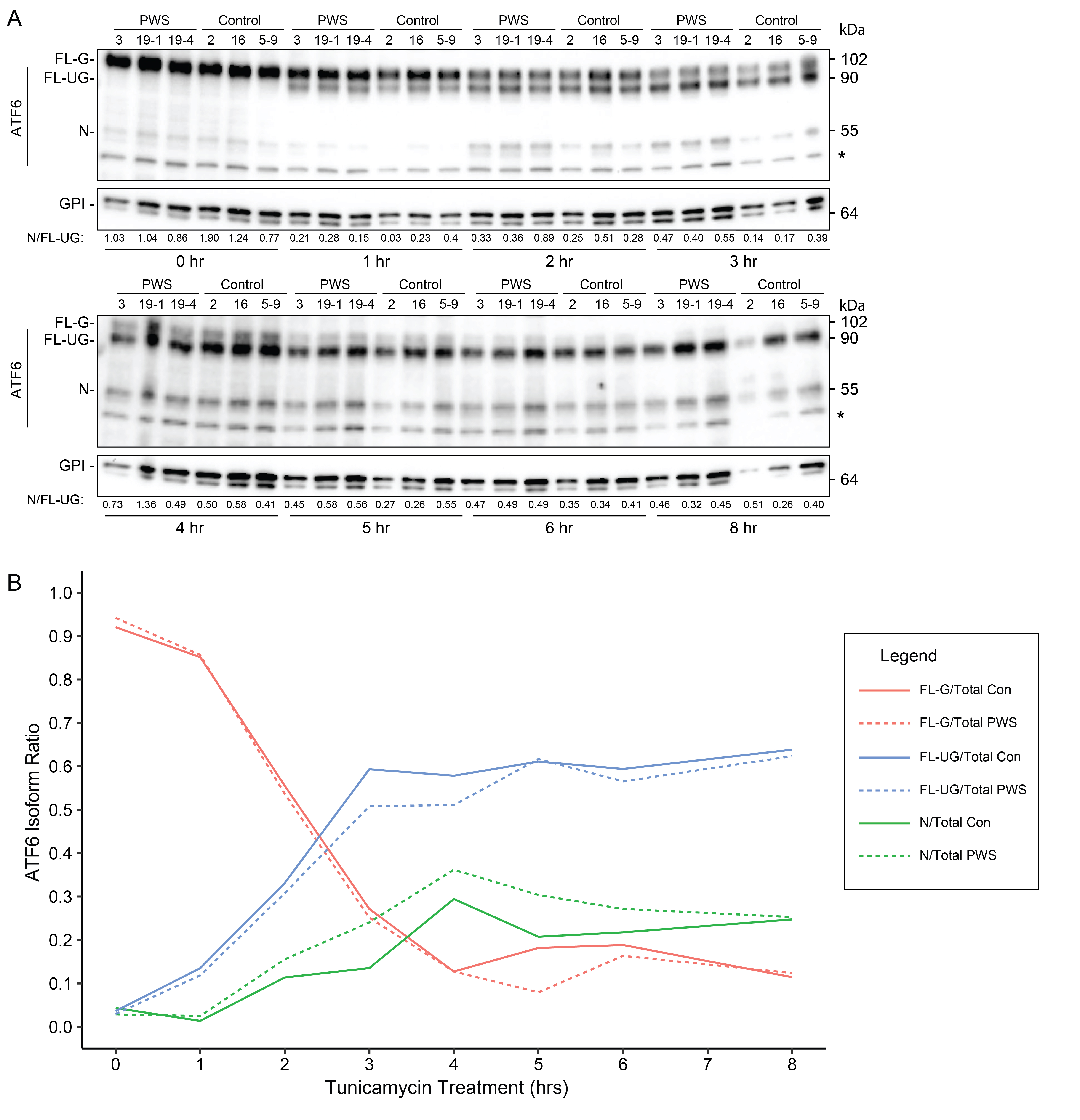

Supplement: S19 Fig — (A) Time-course western blots of ATF6 in PWS and control cell lines treated with tunicamycin for 0, 1, 2, 3, 4, 5, 6 or 8 hours. The ratio of processed nuclear (N) isoform over full-length unglycosylated (FL-UG) is written below each lane. FL-G, full-length glycosylated; *, non-specific band detected by the anti-ATF6 antibody. (B) Relative ratio of each ATF6 isoform as a fraction of the total ATF6 during the time-course of tunicamycin treatment. Line graphs represent mean of three measurements at each time point, except n = 6 using technical replicates for 4 hr and n = 6 using two biological replicates for 5 hr timepoints, for full-length glycosylated (FL-G) over total (Red); FL-UG over total (Blue) and N over total (Green) with control (Con) as solid lines and PWS cell lines as dotted lines. Data indicates that there is a clear trend to more robust processing of FL-UG to N at early tunicamycin timepoints in PWS cell lines. (JPG) [file pgen.1010710.s019.jpg]
