# Supplementary material for: Insulin secretion deficits in a Prader-Willi syndrome β-cell model are associated with a concerted downregulation of multiple endoplasmic reticulum chaperones
Source: PLoS Genet. 2023 Apr 17;19(4):e1010710. doi: 10.1371/journal.pgen.1010710 (PMC10138222; doi:10.1371/journal.pgen.1010710)
Supplement: Attachment — Submitted filename: Responses to PLoS Genetics reviews_All-Points_Final.doc [file pgen.1010710.s030.doc]

**Appendix. Point-by-point responses to reviewers of PGENETICS-D-22-00722 (article for *PLoS Genetics*)**

Note that all points raised in the cover letter from the Editor have been addressed in the attached manuscript resubmission Cover Letter, while specific responses to each point raised by the reviewers are presented in purple font in our detailed responses below. A reference cited herein by name and year is listed in a Reference Cited section below, while references “ref. n” are as cited by number (n) in our manuscript. In our responses to reviewers, line numbers refer to the fully revised “Clean” version of the manuscript, while a “Marked” version showing all revisions from our original submitted manuscript using “Track Changes” is also submitted.

**Author Responses to Questions and Points from the Reviewers**

**REVIEWER #1:** The manuscript by Koppes et al describes experiments in the INS-1 rat beta cell line seeking to understand whether there is an underlying beta cell defect in PWS. As the authors point out, the hormonal defects in PWS are complicated, with potentially interacting defects at the level of hypothalamus, pituitary and end organ hormone producers such as beta cells. In human PWS, the most prominent physiology is hyperphagic obesity and GH/pituitary deficiency. Diabetes sometimes occurs but is thought to be secondary to marked early onset obesity. Although this manuscript contains a large amount of carefully performed work, this reviewer is not sure that the results shed meaningful light on the biology of PWS

The reviewer presents an incomplete view of the pathophysiological basis of PWS, that the disorder is due solely to hypothalamic and pituitary deficiencies leading to hyperphagic obesity and GH deficiency. Although there is evidence for neuroendocrine deficits in PWS, no evidence rules out other organ systems having a role in PWS. Likewise, the statement “end organ hormone producers such as beta cells” with respect to PWS is likely inaccurate, as evidenced by our published studies in the TgPWS (transgenic PWS-deletion) mouse model demonstrating an embryonic/fetal origin of pancreatic islet deficiencies including (but not limited to) β-cell deficits with an insulin secretion deficit (refs. 16, 25). Indeed, our current manuscript now importantly demonstrates that this insulin secretion deficit is cell autonomous (i.e., without neural or humoral input) and expands this phenotypic component to deficits in secretion of other hormones by the β-cell, including IAPP (amylin), and neuropeptide Y (Npy). Interestingly, the etiology of diabetes in PWS is complex, thought to be type 2 diabetes (T2D) secondary to obesity (see ref. 3) but the rate of T2D observed in PWS subjects is lower than expected for the degree of obesity and with relatively low insulin levels. Finally, as stated in our **Introduction**, and **Discussion** first paragraph, while we clearly relate our work to the human PWS clinical disorder, the primary rationale for the scientific studies leading to the conclusions of this manuscript relates to extending an understanding of the β-cell deficits that our prior studies have established as a major component of the phenotype in a PWS mouse model (see above); these considerations are further expanded on in response to Q1 below. Finally, the reviewer statement also reflects a misconception that a β-cell defect must relate to diabetes and in PWS this must be secondary to obesity; in contrast, in the TgPWS mouse model prior to onset of postnatal hypoglycemia, we previously described (refs. 16, 25) not only hypoinsulinemia associated with an insulin secretion defect but co-occurrence of hypoglucagonemia (suggestive of a glucagon secretion deficit, but this has not been further studied), an endocrine co-occurrence not found in any other disease condition and attesting to the likely unique metabolic and endocrine nature of PWS.

1. Rationale for the study is weak: beta cell functional deficiency does not seem to be a prominent feature of PWS

Please see above discussion, as the reviewer incorrectly depicts the rationale for the study described in our manuscript. Furthermore, β-cell function has largely been unstudied in PWS in human. In our **Discussion** first paragraph, we highlight the most detailed studies of Dr. Bill Zipf showing that “PWS adults compared to matched obese controls had significantly reduced first- and second-phase insulin secretion during intravenous glucose tolerance tests” (ref. 11), but these studies were performed in the early-1990’s and further studies are needed. Pancreas has never been available to study from PWS subjects (I am trying to work with PWS family organizations to rectify this issue) to assess whether islet and β-cell deficits of the type we demonstrated in the PWS mouse model (ref. 25) occur in PWS subjects. Remarkedly, until we discovered that PWS mice (with inactivation or loss of all PWS-imprinted genes) only survived less than 1 week postnatally because of postnatal onset and progression to severe hypoglycemia (refs. 16, 25), hypoglycemia had never been assessed in PWS subjects. Indeed, PWS infants have episodes of hypoglycemia (refs. 13, 14). Many other clinicians state anecdotally that adults with PWS also have episodes of hypoglycemia, especially at times of stress, but this remains an understudied and under-published aspect of the PWS clinical phenotype. Further, in independent work that we are writing up for publication, we found that a PWS pig model also had episodes of hypoglycemia, attesting to the conservation of this PWS endophenotype across mouse, pig, and human. Finally, in the last section of our **Discussion** of the current manuscript, we reflect on the relevance of our rodent model observations to expand on the undisputed facts that “Maintaining glucose homeostasis relies on exquisite coordination between secretion of the opposing hormones insulin and glucagon from pancreatic β- and α-cells, respectively, in response to changes in blood glucose”, and relate the work in our current manuscript (and previously published work) to this to justify the importance of further study related to our novel discoveries related to the PWS β-cell (INS-1) rodent model.

2. Studies only performed in a rat transformed insulinoma cell line are relevant to a human condition. Validation in the mouse model would increase relevance but the perinatal lethal hypoglycemia in the mouse model already differs strongly from the human condition

Unfortunately, the latter part of this assertion is just not true; as discussed above, with respect to the mouse model the perinatal lethality is due to severe hypoglycemia (refs. 16, 25), while hypoglycemia is also an endophenotype in human PWS (refs. 13, 14) but an aspect that is understudied. Indeed, episodes of hypoglycemia may well be a major factor contributing to neurodevelopmental deficits in PWS, which should be studied! We were able to keep our PWS pig alive through hypoglycemia episodes by specific treatment regimens (manuscript in preparation); the typical gavage feeding in PWS infants may help in this regard in the human case. Interestingly, recent animal studies have shown that hypoglycemia induces hunger and food intake (new ref. 107; Aklan et al. 2020), which supports a hypothesis that hypoglycemia may explain or contribute to hyperphagia and obesity in PWS. We have now cited this work in our current manuscript, on page 28, line 577. Indeed, in the PWS mouse model we previously showed that excessive ghrelin levels inversely correlate with the degree of hypoglycemia (ref. 16), drawing another likely parallel with the endocrine phenotype of PWS. Thus, this is a topic that begs for further study and may lead to fundamental new insights into the complex, pleiotropic pathophysiological basis of PWS.

Furthermore, it is important to point out that validation studies in the mouse model have already been done that formed the rationale for the current work (as stated in the **Introduction**), including direct validation of an insulin secretion deficit in TgPWS *vs.* control islets (ref. 25) while indirect support consistent with the potential impact of ER chaperone deficits affecting the insulin secretory pathway arises from our previous *in vivo* studies in the mouse model that showed accumulation of aged insulin in TgPWS β-cells (ref. 25); these points also relate to and are expanded upon below in response to Reviewer #2, Q4.

**REFERENCE CITED** (new ref. 107)

Aklan I, Sayar Atasoy N, Yavuz Y, Ates T, Coban I, Koksalar F, Filiz G, Topcu IC, Oncul M, Dilsiz P, Cebecioglu U, Alp MI, Yilmaz B, Davis DR, Hajdukiewicz K, Saito K, Konopka W, Cui H, Atasoy D (2020) [NTS catecholamine neurons mediate hypoglycemic hunger via medial hypothalamic feeding pathways.](https://www.ncbi.nlm.nih.gov/pubmed/31839488) *Cell Metab* 31(2): 313-326. PMID: 31839488.

**REVIEWER #2:** In this study, the authors sought to understand the cell-autonomous mechanisms that may underlie beta cell dysfunction observed in Prader-Willi syndrome (PWS), which includes hypoinsulinemia as a known feature. Using CRISPR, the authors deleted a genomic segment associated with paternal gene imprinting that defines PWS in a modified INS1 beta cell line. The authors conclude that PWS limits expression of ER chaperones, which thus limits the beta cell’s insulin production capacity. This is an interesting concept that emphasizes the field’s increasing understanding of how ER function plays a direct role in the pathogenesis of many forms of diabetes and may ultimately reveal novel mechanisms for modification of ER function. The study benefits from non-biased approaches (e.g. mass spectrometry and RNA sequencing) to broadly evaluate the consequences of the PWS in beta cells. However, there are some limitations that question the broader application of these findings to a clinical setting. These include the sole use of the INS1 cell line, which does not precisely reflect the biology of islet beta cells (in part for the expression of insulin genes from multiple species) and the lack of investigation into whether the findings here would be replicated in a more physiologic setting. The writing overall is organized and easy to understand, though the precise mechanism underlying the observations remains speculative.

We thank the reviewer for recognizing many of the salient and novel aspects of our manuscript, and that the manuscript is well written. Each of the issues raised by the reviewer is addressed under the specific questions/comments below, from which we demonstrate that limitations are minor, that the majority of studies in model systems (animal or cellular) are not immediately directly relevant to a clinical setting but still important and critical, the rationale for use of β-cell models, and that we have already replicated a significant component of the current study in a physiological setting in an *in vivo* mouse model.

Major Issues:
1. The mechanism proposed to explain defects in PWS beta cells seems to be downregulation of ER folding proteins that subsequently impairs proinsulin production/maturation. A final schematic (with known and unknown mechanistic links) would improve clarity. The overarching question, however, seems to remain unanswered: how does reduction in critical ER proteins limit insulin production, in the absence of other evidence of ER dysfunction (e.g., overt activation of the UPR, ER dilation, or insulin misfolding/aggregation)? Can these findings be replicated by directly reducing expression of some of the key ER proteins in question?

Our manuscript details extensive data showing that PWS INS-1 cell lines, as an experimentally highly tractable model for PWS β-cells, have a cell autonomous deficiency in production and secretion of peptides secreted from β-cells, including insulin, IAPP (amylin), and Npy, accompanied by an associated deficiency in production of ER chaperones. We have rewritten the first paragraph of our **Discussion** to better reflect the specific key observations from the work. The two subsequent sections of the **Discussion** then expand upon these themes and relate our discoveries to the current knowledge in these fields. Indeed, this includes on pp 21-22, lines 433-443, relating findings from various chemical and/or genetic approaches to knockdown *Hsp90b1* (GRP94) levels (ref. 67) and knockdown *Hspa5* (GRP78) levels (refs. 68, 69), each of which affect insulin production and secretion and leads to our conclusion that knockdown of the levels of the 10 ER chaperones that we observe unsurprisingly thus results in deficient insulin secretion, points that the reviewer failed to consider in suggesting directly reducing expression of some key ER proteins in question (i.e., this has already been done). We go on in the subsequent **Discussion** section, pp 22-23, lines 447-463, to relate additional published studies on reductions in specific ER chaperones to compensatory changes in other ER chaperones (refs. 70-73). At this time, we do not have a known mechanism that leads from the PWS genetic defect to these peptide hormone and ER chaperone deficiencies, either from our data or the literature, and determining a mechanism is well beyond the scope of this manuscript and will require much additional funded work (indeed, most PWS genes are of poorly understood function and mechanisms are unknown leading to any disease phenotypic component). Critically, our third **Discussion** section, has been renamed “Mechanisms for …” and has been rewritten to better formulate potential mechanisms and to present an alternative mechanistic hypothesis (new paragraph on pg. 25, lines 508-525). Indeed, we agree with many of the reviewer’s questions, but these require future studies beyond the scope of the current manuscript. Last, the reviewer suggests a final schematic may improve clarity of potential mechanisms; at this stage, we feel that our revised **Discussion** sufficiently evaluates mechanistic hypotheses and that future work beyond the scope of the current manuscript is needed to make an accurate schematic on mechanisms.

2. The presence of multiple insulin genes in this cell line may confound the results, as the changes in insulin gene or protein expression may be different than that in native islet beta cells. More importantly, there is little discussion regarding the presence or absence of higher molecular weight forms of misfolded/aggregated insulin in the PWS cells. In the setting of greatly reduced ER chaperones, one might hypothesize that insulin misfolding and aggregation within the ER would be a likely contributor to impaired insulin maturation and cell dysfunction. Were such forms found in the mass spec or Western blot data? And if not, what would be the explanation?

The vast majority of the insulin transcripts and hence secreted insulin from the rat INS-1 cell model are from rat *Ins1* (85.5% or 90%, for the control *vs.* PWS lines) with rat *Ins2* contributing only ~ 1.2% or 1.6% (for control *vs.* PWS), the mouse *Ins2*-mCherry transgene contributing ~ 12.8% or 8.8% (for control *vs.* PWS) and the human *INS* transgene contributing negligibly (0.02% or 0.04% for control *vs.* PWS) [see **Supp. Info S16M Fig** for the RNA-seq mRNA levels of each]. Hence, given that for both control and PWS INS-1 lines there was ~ 10% contribution of transgenic insulin gene expression to the total insulin mRNA pool it is unlikely to be the major insulin isoform driver of protein folding load as detected by the UPR. While a reduction in insulin (the secretory cargo) could modulate ER chaperone levels via an undefined feedback loop (as per our **Discussion**, pp 24-25) the simpler explanation and the one we favor is that the primary genetic defect (PWS deletion) in our PWS INS-1 cell lines drives changes in levels of mRNAs encoding secreted peptides and ER chaperones and their corresponding changes in protein levels directly contributes to the observed deficits in insulin secretion.

To the reviewer questions regarding the potential for insulin aggregation, the processing of CP-mCherry from pro-mINS2-mCherry is evident in the SDS-PAGE (with β-mercaptoethanol reducing agent) western blot (**Fig 5A,C**), however larger higher molecular weight aggregates were not detected nor were they detected by mass-spec proteomics analyses. Putative aggregation is also refuted by the lack of UPR or ER-stress activation in the control or PWS INS-1 lines under DMSO (untreated) conditions (**Fig 6A-F**) as would occur if either endogenous insulin or mCherry-C-peptide was misfolding or aggregating. Our confocal studies (now removed; see below) did not utilize subcellular colocalization markers for ER, Golgi, lysosome, or insulin granules so we could not fully assess trafficking and whether aggregates were being formed, studies beyond the scope of this manuscript. That being stated, for the past 20 years various versions of insulin C-peptide fluorescent biomarkers have been used in cell culture and *in vivo*, including by us, without aggregate formation, nor have UPR or ER-stress responses been observed in those models. Also see Minor issue #2 below for discussion of the detected insulin forms (**Fig 5A**).

3. It is quite striking that the PWS cells do not show increased UPR activation at baseline with such a significant reduction in ER chaperones. Is there a difference in expression of UPR sensors, or other reason to help explain a blunting in response? Additionally, it is unclear if the slightly more rapid (but still limited) activation of UPR pathways in stressed PWS cells has clinical relevance, particularly as this was only observed following treatment with potent ER stress activators (thapsigargin and tunicamycin). Does inhibition of UPR signaling improve the cells’ ability to produce mature insulin? Was there an attempt to evaluate the response to more physiologic stressors in these cells, such as palmitate or high glucose?

This hypothesis of the reviewer for increased UPR activation at baseline would only be true if there was ER stress in the PWS cells at baseline, and activation of the UPR would then have the effect of increasing expression of ER chaperones. We do not observe ER stress within PWS cells either at baseline (i.e., standard cell culture conditions of 7.5 mM glucose) or within the 30-minute test time of high glucose conditions, itself a stressful physiological state. Only on treatment of PWS cells with severe ER stress-inducing chemical compounds (thapsigargin or tunicamycin) do we see activation of the master sensor pathways of the UPR. Indeed, it is surprising that the reviewer asks whether there is “a difference in expression of the UPR sensors”, as we demonstrate that there are no changes in these 3 pathways, by proteomics (**Fig 3**; and our dataset deposited at the ProteomeXchange Consortium) and by RNA sequencing (**Fig 4**, **S12A Fig**, **S1 Table, S2 Table**: and our dataset GSE190334 deposited at NCBI GEO), and by directed studies (**Fig 6**) of activators of the 3 pathways, as represented by “splicing” of *Xbp1* transcripts, by phosphorylation of eIF2α, and by cleavage of ATF6 to the nuclear ATF6-N form, where PWS and control cell lines are indistinguishable at baseline (**Fig 6**).

4. This manuscript would be greatly improved if the main findings were formally evaluated in a more physiologic model, such as tissue sections or isolated islets from TgPWS mice. In this draft, the explanation for differences with previous findings in TgPWS mice is insufficient (Discussion lines 409-419).

The major point here on *in vivo* evaluation in mice is equivalent to Q2 of Reviewer #1 above, as briefly addressed in that response, while we expand on these points here in greater detail for full clarity. Thus, one of the two major findings in the current manuscript is demonstration that the insulin secretion deficit in PWS rodent models is β-cell autonomous (i.e., not involving neural or humoral input), also extended through our detailed proteomic studies demonstrating that all major secretory peptides of INS-1 cells (insulin, IAPP/amylin and Npy) are deficient in secretion. As the major rationale for the current work (see **Introduction**), the previous work showing an insulin secretion deficit in TgPWS *vs.* control islets (ref. 25; reproduced as **Fig. 1A** below) provides direct validation of the insulin secretion deficits now observed in our PWS ***vs.*** INS-1 β-cell model (Koppes et al., current manuscript) along with indirect support for the potential impact of ER chaperone deficits affecting the insulin secretory pathway due to our previous *in vivo* studies in the mouse model (cont.)

**Fig. 1. Previously published data from TgPWS *vs.* wildtype (WT) mice at postnatal day 1 (P1), an age at which blood sugar levels are normal (ref. 25). (A)** There is reduced insulin secretion for TgPWS ***vs.*** WT mice using purified islets cultured *in vitro* at both low (2 mM) and high (20 mM) glucose (ref. 25). **(B)** Insulin secretion *in vivo* in WT and TgPWS mouse islets, based on an Ins-C-Timer transgene for which fluorescence changes from green to red over 24 hr to report on the time since co-synthesis of insulin and the C-peptide-Timer fusion (ref. 25). At P1, β-cells in WT show mainly green fluorescence of newly synthesized insulin, whereas β-cells from TgPWS show mostly red fluorescence indicating a delay in secretion of insulin (ref. 25).

showing accumulation of aged insulin in TgPWS β-cells (ref. 25; reproduced as **Fig. 1B** above). Although indirect, these published observations support our new description of a significant ER chaperone deficit in the PWS INS-1 β-cell model (Koppes et al., current manuscript).

Additionally, the reviewer suggests that our explanation (lines 409-419 of our original manuscript) for differences observed between the *in vitro* RNA sequencing data in the present manuscript and the previous findings from microarray studies of RNA expression using pancreas from the TgPWS *vs.* WT mouse model (ref. 25) is insufficient. While our previous work suggested that the upregulation of mRNAs encoding secreted peptides and other secretory polypeptides related to an apparent physiological compensatory mechanism (ref. 25), we have now expanded on that (see lines 419-429 of our current manuscript) to indicate that the INS-1 model reveals a contrasting cell autonomous mechanism (i.e., without neural or humoral input).

Minor Issues:
1. Figure 3 readability would be greatly improved with larger text sizes and the addition of headings for the graphs in Β-D. Also, Figure 3A and the legend should be modified to describe B through D in order.

We agree and have modified **Fig 3A,B,C,D** accordingly. The authors thank the reviewer. In addition, as the text within **Fig 4A** was even smaller, we have adjusted to improve readability of that figure; these and all other changes to **Fig 1-6** in the manuscript are summarized below in a separate “Additional Changes” section.

2. In Figure 5A, there are several unlabeled bands. What insulin forms would these be? Does this insulin antibody have equal affinity to the different species of insulin?

In **Fig 5A**, the unlabeled bands are almost certainly Pro-mINS2-mCherry proteolytic fragments not containing the anti-mCherry epitope, as not detected in **Fig 5C**. A note to this effect has been added to the **Fig 5** legend, to make this clear. This insulin antibody has equal affinity to rat, mouse, and human insulins; all antibody details are presented **in S9** **Table** including the Supplier and Catalog No., from which all details on the specificity and affinity can be obtained.

3. The electron microscopy data (Supplemental Figure 17) would be improved with a formal evaluation of insulin granule number and size, though it appears that the current images do not provide adequate representation of granule morphology. Is such data available?

By comparison with the literature and **Fig 5A**, INS-1 cells produce much less native insulin and pro-insulin than the INS1-E cell line (although INS-1 cells produce more pre-pro-INS), which makes a formal evaluation of insulin granule number and size not possible from the available electron microscopy data. This is an interesting question for future studies beyond the scope of the current manuscript. We have added several sentences to the legend of **Supp. Info S17 Fig** addressing this issue and further highlighting the aspect already stated in our main manuscript (pg. 16, lines 330-332) that this data shows visually normal organelles such as mitochondria and rough ER.

4. Supplemental Figure 18E-F seems to show a larger cohort of cells in these PWS lines with reduced mCherry expression, in disagreement with authors’ conclusion that C-peptide-mCherry expression is unchanged in PWS cells (Figure 5B, E). Does the construct only show mCherry fluorescence after insulin processing (in contrast to the mCherry found in both proinsulin and cleaved C-peptide on Western blot)? This should be clarified, or more representative images chosen.

Please see the response to reviewer #3, Q16, that details an equivalent comment.

**REVIEWER #3:** This manuscript investigates the cellular basis of reduced insulin secretion in Prader-Willi syndrome (PWS) by generating several independent INS-1-derived cell lines to model PWS. These PWS model cells have reduced insulin secretion and reduced levels of insulin and several other secreted peptides. A possible mechanism is suggested by transcriptomic and proteomic analyses, demonstrating the reduced expression of a number of endoplasmic reticulum (ER) chaperones that may be important for proper folding of insulin; reduced levels of chaperones may lead to reductions in insulin levels, though it should be emphasized that this is just a model and hasn’t been tested. The experiments are performed well and the data are solid and convincing, though the effect sizes are of rather small magnitude and it is unclear how important the small effects seen here are to the pathophysiology of PWS. The extensive and rigorous molecular characterization of the mutations in the PWS model cell lines is a particular strength, and the fact that several independent PWS and control cell lines are generated increases the confidence in the results. The proteomic and transcriptomic datasets generated in this work are important contributions to the field. We have a number of relatively minor critiques, many related to the writing and presentation of the work.

We thank Dr. Michael Ailion and Dr. Chau Vuong who signed their names to the review for their detailed and thoughtful consideration of our manuscript and value their input to further improve our manuscript. We are grateful that they appreciate the extensive and rigorous molecular analysis of the genome edited mutations, especially as few studies have successfully generated model systems with ~ 3 Mb deletions. We agree that the mechanism(s) for the associated reductions in secreted peptides and ER chaperones remains to be tested, but importantly, the molecular and cellular mechanisms along with potential therapeutic implications are the basis for a new 5-year NIH RO1 application and are beyond the scope of the current already very extensive and detailed manuscript.

Specific comments:

1. Though the data appear to be solid, virtually all of the effects are of small magnitude, < 2 fold (e.g. insulin secretion, altered expression of chaperones at both the mRNA and protein levels, ER-stress sensitivity). This is fine, but is not readily apparent from the way the paper is written (e.g. line 400, where insulin secretion is described as “dramatically reduced” or elsewhere where effect sizes can only be gleaned by careful examination of the figures). More transparency and explicit discussion of the effect sizes would be helpful. For instance, it would be helpful to compare the effect sizes seen here to those of PWS mouse models and human patients.

Although the reviewers suggest our study identifies relatively small magnitude effect sizes, this consideration depends on what this means in context. Our significant effect sizes are on the order (1.5- to 2-fold) typically seen in genetic disorders with chronic, long-term disease (e.g., PWS, Down’s syndrome, etc.). It is quite remarkable that most INS-1 secreted peptides are significantly reduced from PWS cells in analyzing insulin secretion (**Fig 2**) and secreted peptide proteomics (**Fig 3D**). Further, on the deficits in many individual ER chaperones, the combined deficits for all such components in a single functional pathway would be expected to have a significant biological effect; this point is discussed in much more and specific detail in response to the next question (#2). Rather, larger effect sizes occur in acute disease states, such as cancer, conversion of pre-diabetes to diabetes, heart attacks, death, etc.

2. The take-home model of the paper is that the effect on insulin in PWS is due to an indirect effect on chaperones. This is a reasonable and interesting model, but given that the magnitude of the downregulation of these chaperones is actually quite small (appears to be less than 2-fold for most or all of them), it seems possible that some other mechanism is at play and this may be a red herring. It is unclear whether such a modest decrease in multiple chaperones would produce the observed effects on insulin content and secretion, though it is an interesting question for future work. However, it would also be nice to see the full lists of proteomic and transcriptomic data presented as supplementary tables for interested readers so possible alternative targets can be more easily explored.

We have rewritten parts of the **Abstract** and especially the **Discussion** to highlight that while our data combined with literature considerations suggest the model that the “broad ER chaperone deficiency in the PWS INS-1 model as a primary abnormality that contributes to diminished insulin secretion” (lines 441-443), we do not rule out, and our presented evidence rather includes, models in which PWS cells also have primary deficits in production of secreted peptides in combination with the ER chaperone deficits. It should be noted, as we discuss in detail in our **Discussion** (lines 430-485) that the levels of ER chaperones are exquisitely controlled for cellular homeostasis and in secretory cells, where the ER is already under stress, that even minor disruptions can lead to acute levels of ER stress. The level of downregulation we detect for each ER chaperone is not minor, indeed even minor perturbations in ER chaperone levels lead to compensatory expression in other ER chaperones; in particular, for GRP78 *vs.* GRP94, the two major β-cell ER chaperones. As we cite on page 22, lines 448-454, even in the *Hspa5* (encoding GRP78) heterozygous (+/-) knockout mouse model this 50% decrease in gene dosage (i.e., 2-fold, the same order of magnitude as dysregulation in our study) is sufficient to require compensatory increases in GRP94 expression (refs. 70, 72), and in the same section we cite additional studies in other models that reflect equivalent observations on compensation among ER chaperones (refs. 71, 73). This is for alterations of single ER chaperones, whereas as we state clearly (lines 456-463) our description of widespread downregulation in PWS cells for at least 10 ER chaperones expressed in β-cells, including GRP78 and GRP94 that are the most highly expressed, prevents any such compensatory increases in expression. The level of ER chaperone dysregulation that we describe is unprecedented, and based on the literature we discuss, would be expected to have a major biological consequence.

Finally, as noted elsewhere in other responses, all the transcriptome and proteomics datasets are available from repositories no later than the time of publication. Furthermore, every significantly differentially expressed mRNA (**S1 Table**, **S2 Table**) and small RNA (**S3 Table**, **S4 Table**, and **S5 Table**) are presented in the **Supp. Info**.

3. It is concluded that PWS cells are unable to compensate for decreases in chaperones because many chaperones are simultaneously downregulated. This argument does not make a lot of sense to us, and would seem to depend on the mechanism of compensation, which is not further described here. For example, if chaperone genes are transcriptionally downregulated in PWS mutants, what precludes an independent compensation mechanism from simply turning transcription back up, unless the PWS genes are important for the compensation process itself? It would help to present more about what is known about this compensation mechanism, and whether it occurs transcriptionally or posttranscriptionally. A small decrease in many chaperones does not inherently seem to preclude a possible compensation mechanism.

Please see the previous response to question #2. The mechanism of compensation is not known and is an important area for further research, not just for our work but for that by other investigators in the ER chaperone and related fields. We do agree, however, that our section of the **Discussion** (pp 24-26) on mechanisms was not sufficiently well explained and have rewritten much of this section. In particular, we point out (lines 493-494) that it is unknown as to whether the reductions in RNA levels for genes encoding ER chaperones and peptide hormones occurs by a transcriptional, mRNA stability, or mRNA processing mechanism.

4. The paper is rather difficult to read with lots of jargon and a poor narrative flow. The reader has to do a lot of work to figure things out on their own without much help. Several examples of this are provided in the next few comments (#5-8).

We will address specific comments below in #5-8; we do not feel that our manuscript uses jargon but rather uses terminology represented in the mammalian genetics and genome editing fields and that this is reflected in use of citations from the literature. To improve the narrative flow, we have broken the previously longest **Results** section on RNA sequencing into two subsections, each with a new subtitle; otherwise, in both **Results** and **Discussion** we have broken each into subsections with descriptive subheadings and include frequent citation to literature and to our presented data (in **Figures** or **Tables** in the main manuscript and **Supp. Info**).

5. Some genes or proteins come up quite suddenly without mentioning their functions or significance. For example, in lines 96-101, SNRPN, SNORD116 and SNORD107 have not been introduced yet as PWS genes, which makes the subsequent conclusion confusing that PWS genes function in beta cells.

These gene names arise after beginning the paragraph stating that the PWS-imprinted domain comprises 10 genes conserved in mammals “encoding lncRNAs, snoRNAs, miRNAs, or distinct proteins” (lines 90-93). The sentences that follow make clear that the entire paragraph is discussing PWS genes and relevance to endocrine cells; relevant information on all these genes comes from all the references cited in the paragraph, or a quick search of gene databases will indicate what each encodes. However, it is beyond the scope of this manuscript to review the structure and putative functions of each PWS-imprinted gene.

6. In several places in the proteomics and transcriptomics sections, there are long lists of genes or proteins with very little context to orient the reader. It is hard for the reader to make much of these lists, and some guidance as to why they are considered worth pointing out or short take-home messages in these sections would be useful. For just a couple examples of this see lines 287-288, lines 267-268.

We disagree, as in every instance of these sections we provide the written context along with citing the relevant **Figure**, **Table**, or literature citation. This includes for the two suggested examples, where we specifically state what each brief “list” relates to. Adding further information would only bloat the manuscript and lose context for each such point.

7. The description of engineering PWS INS-1 cells is quite hard to follow. Figure 1B is not very intuitive. These sections demanded a lot of work from the reader, much of which required looking at supplementary figures to understand the main Results sections. As many readers may not look at these figures, it would help to make this section more accessible.

The genome editing as described on pages 8-11 (text), **Fig 1**, and **Supp. Info S1 Fig** through **S9 Fig** provides a comprehensive description of the detailed and complex genome engineering. In preparation of the manuscript, we placed critical data in **Fig 1** for readers to understand the basis of the process and nature of the cellular model, with complementary and complete information in the **Supp. Info** for those readers who were interested in following such a detailed characterization [note that in our opinion most genome editing papers only incompletely and often poorly characterize or detect mutations]. The information is readily accessible from the **Supp. Info**, for interested readers, while those interested in the biological outcome of the model can move more readily on from **Fig 1** to the rest of the manuscript.

8. The rationale for performing RNA-seq of small RNAs is not provided. This section ends up interrupting the main narrative and feeling tangential.

Please see lines 262-264 where we provide the rationale for this aspect of the study. Small RNAs are a critical part of the PWS-imprinted domain, and likewise are major regulators of β-cell regulation (e.g., MiR375). Furthermore, the data provided allow a direct comparison of the rat INS-1 β-cell model to a human β-cell model, as we note on lines 269-272. This is important to confirm that the INS-1 model is a valid cellular model of β-cell function.

9. Since PWS cells have altered levels of many proteins, it is unclear whether the total protein content is a good parameter to use for normalization of insulin secretion.

PWS cells have significantly altered levels of very few proteins, and the majority of these are proteins within only two biological pathways, ER chaperones and secreted peptides (see **Fig 3** and all three proteomics database submissions to the ProteomeXchange Consortium via the PRIDE partner repository). One sentence previously stated “… accompanied by widespread alterations in mRNA levels most notably encoding secreted peptides and ER chaperones” was ambiguous and we have rewritten this sentence to correctly state “… accompanied by alterations in levels of mRNAs encoding numerous secreted peptides and ER chaperones” (page 17, lines 337-338). No protein markers or regulators of cell death or cell proliferation are differentially expressed by either proteomics or whole genome RNA sequencing for PWS cells. Therefore, total protein content is a perfectly appropriate parameter to use for normalization of insulin secretion. Furthermore, insulin secretion assays are notoriously difficult at the practical level due to the large numbers of washes through the different steps of the assay that can lead to experimental losses of cells; this is controlled for by utilizing very careful handling steps, by performing the assays with a large n (see **Fig 3** legend) and by normalizing to protein content as a more reliable measure than cell count.

10. It would help to see the unnormalized raw data for the insulin secretion experiments. Figure 2 shows pooled data from several cell lines. It would also be helpful to see the data for each line separately in a supplementary figure.

This is a valuable comment based on which we redrew **Fig 2** to present each individual data point for each cell line. In addition, all the pooled data and all the individual cell line data are presented in two tabs of the new **S1 Data**, in which all the numerical data and statistical analyses are presented. The authors thank the reviewer.

11. It is not stated how many times proteomic and transcriptomic experiments were replicated. It is stated that each was performed on three control and three PWS cell lines, but it is unclear if each line was tested just once. It seems likely that the data depicted in the figures are pooled from the different lines though this is not stated explicitly. More clarity on these points would be useful. Separate figures for unpooled data of each cell line would also be useful in the Supplement so that variability between lines can be seen.

The proteomics (on three different cellular and secreted fractions) and the transcriptomic (RNA-seq) experiments were each performed a single time and not replicated for this manuscript [see # below]; as I’m sure can be appreciated, these experiments are very expensive, and it is not possible to replicate. Furthermore, the primary goal of these ‘omics experiments is to identify candidate proteins and RNAs that are differentially expressed, and then to use follow-up experiments (westerns for the protein; RT-ddPCR for the RNAs) on independent biological and experimental replicates to verify biological significant differences between the disease state (PWS) and control cells. We stated clearly in **Methods** for the Proteomics that each of the 3 control cell lines (5-9, 2, 16) and 3 PWS cell lines (3, 19-1, 19-4) were utilized, and have now added this same statement to the RNA-seq section of the **Methods** and to the **Fig 3** legend. Additionally, **S1** **Table** through **S5** **Table** presenting all the significant data on differentially expressed genes from different analyses of the total RNA-seq experiment (**S1** **Table** and **S2 Table**) and the small RNA-seq experiment (**S3 Table**, **S4 Table**, **S5 Table**) present the individual cell line data for all 6 cell lines, making very clear what was done, and which cell lines comprised the PWS and control groups.

[[# In confidentiality, we have replicated the RNA-seq results presented within the current manuscript in an independent dataset performed solely as a control sample, under different cell culture conditions as part of another project, work that is ongoing and will be the subject of a future manuscript.]]

12. The study emphasizes the deficits in secreted peptides and ER chaperones but doesn’t provide an explanation for proteins that are increased. A number of neuronal active zone proteins are reported to have increased expression at the mRNA level, but for most it is unclear whether this effect extends to the protein level (only CHGB is labeled in Figure 3). The possible relevance of these changes is also unclear. It is pointed out that many of these proteins may play a role in insulin secretion, but it is unclear why potentially increased levels would lead to the decreased secretion observed in PWS cells unless these factors are negative regulators of insulin secretion (though that seems unlikely given their neuronal functions). Thus, the relevance of these results is unclear.

The reasons for emphasizing the downregulated secreted peptides and ER chaperones are because these were the most significant alterations, were all confirmed by multiple experimental approaches, and biological causation and role can be understood and/or proposed. In contrast, fewer upregulated mRNAs and almost no proteins at the proteomic level were detected, only a subset of the potentially upregulated mRNAs were confirmed, and as the reviewers point out the biological significance of the latter are not necessarily clear, largely as less is known about potential roles for these. It is important for completeness of description of the results to point out these data but in a succinct way; furthermore, no publication of this type with genome-wide proteomic and transcriptomic data can be expected to (or ever has) understood every potentially differentially expressed gene or protein.

13. It would be helpful to explicitly state in the Results how many of the genes with reported changes in RNA levels were validated and were not validated by RT-PCR experiments (e.g. line 308).

The vast majority and all the most important DEGs were validated (**Fig 4D** = 10, **Fig 4E** = 4, **Fig 4F** = 2, **S12B Fig** = 10, **S12C Fig** = 4, and two non-DEGs were validated, **S12E Fig** = 2; total = 32 DEGs validated). All PWS-imprinted genes were also validated (including for additional transcripts not represented by the “10 conserved genes” in this domain), for a total of ≥ 42 DEGs validated. Only 8 potential DEGs were not validated (**S12D Fig**), although the latter tended towards the down- or up-regulated levels suggested by RNA-seq without statistical confirmation (and were less significant in the RNA-seq).

14. It is unclear whether it is standard practice to use an anti-KDEL antibody in Western blots to specifically identify GRP94 and GRP78, given that this antibody would be expected to recognize many proteins. If so, it would be helpful to cite other articles that validate this method or state the same thing.

Use of the KDEL antibody to detect primarily GRP78 and GRP94 is a standard approach in many hundreds of published articles. It specifically detects the KDEL epitope at the C-terminus of proteins retained in the ER and represents a signal for the KDEL receptor in the Golgi for retrograde transport back to the ER. While additional proteins with a KDEL C-terminal motif can be detected in some cell types or on long western blot exposures, in insulinoma β-cell models such as INS-1 cells the major ER chaperones are GRP78 and GRP94 (this can also be seen clearly in the proteomics, **Fig 3B**). Given the ease of finding such standard information in the literature or commercial antibody product pages, we have not further expanded the reference list.

15. Electron microscopy images in Fig S17 show one picture of each cell line, leading to the conclusion that PWS cells have normal ultrastructure. It is unclear what criteria were used to make this apparently subjective conclusion (no quantitative data are presented). Also, there is no mention of how many cells and sections were examined.

A representative image for each cell line is depicted in **S17 Fig**, but many images per cell line were assessed, by co-authors Dr. Donna Stolz, Dr. Simon Watkins, and Dr. Peter Drain; all highly experienced cell biologists particularly with β-cells. This was a minor component of the study, presented in **Supp. Info** to support lack of any marker of cell death (mitochondria ultrastructure) or ER stress (rER ultrastructure). To further enhance what was done and the relevance, we have added several sentences to the **S17 Fig** legend.

16. Fig S18: confocal cell images are difficult to assess. It would be helpful to zoom in on one cell for better comparison. As with EM data, it is unclear what criteria were used to compare the PWS cells to control and no quantitation is provided, nor is there mention of number of cells examined.

We agree that the confocal images are difficult to assess and would require many additional experiments to properly assess the trafficking of fluorescent biomarkers through the cellular secretory apparatus of PWS *vs.* control cells; therefore, we have chosen to remove this data and the associated **Methods** section from the manuscript. Indeed, there are newer fluorescence timer biomarkers that we plan to use in future studies, beyond the scope of this manuscript.

17. It would be helpful to provide the numbers of the specific PCR primers used in Figs 1, S1 & S4.

All this requested information is detailed in columns of **S7 Table** and **S8 Table**. As each of these figures and figure legends is already large and complex, we would prefer not to further clutter the figures (and legends) with information that we have made readily available in tabular form in the **Supp. Info**.

18. Figs S13B & C are hard to read. They are small and blurry, even when enlarged. The meaning of green boxes in Fig S13C is not explained.

Note that this is now **S14 Fig** for the resubmitted manuscript. Our original submission included the use of JPEG images imported into Word and then submitted as a PDF; for this revised manuscript, we have uploaded higher quality JPEG images (in RGB format) which greatly improves the quality of the images. We added an explanation of the green boxes to the legend of **S14C Fig**. Most importantly, on request from the *PLoS Genetics* team due to copyright issues, we have completely remade **S14B,C Fig** to remove any copyright issues.

19. The bioinformatic analysis of transcription factor binding sites shown in Fig S14 is not described in the Methods. The legend to this Figure has a number of undefined acronyms.

Note that this is now **S15 Fig** for the resubmitted manuscript. The figure legend states each online database program that was used for each analysis, and these (and the underlying acronym) are readily accessible online and well known to the bioinformatics field. Every other abbreviation is defined in the figure legend.

20. Line 154: it is unclear what is meant by “biparental DNA methylation.” This was confusing because we thought it is only the maternal allele that is methylated. Or is this altered in cell lines? Related, it would be helpful to better describe the nature of the two alleles in the cell line because one does not usually think of the chromosomes in a cell line as being of maternal or paternal origin; are methylation and imprinting retained in the cell line?

Biparental DNA methylation is a term regularly utilized by the epigenetic community and applies specifically to genomic imprinting. In the diploid mammalian genome, the DNA methylation state of the two alleles in a cell can have the same state (biallelically methylated or unmethylated) or be differentially methylated and unmethylated. Imprinted genes inherit mono-allelic differential DNA methylation patterns from parental gametic alleles. In the case of the PWS-region the maternally inherited allele is methylated at the PWS-imprinting control (PWS-IC) region and transcriptionally silenced, whereas the paternally inherited allele is unmethylated and transcriptionally active. Thus, the term “biparental DNA methylation” implies inheritance of both maternal and paternal imprinted methylation states. For the second part of the reviewer question, a diploid cell line will typically have chromosomes that were originally of maternal or paternal origin. While some cell lines can alter their chromosome number, epigenetic state, or convert to uniparental disomy, many cell lines retain all epigenetic features including for genomic imprinting, X-inactivation, developmental stage (e.g., for embryonic, fetal, or adult globin gene expression). The imprinting and biparental DNA methylation of the PWS domain is conserved in INS-1 cells, as indicated in the **Results** section on derivation of the cell lines. To avoid confusion, and as recommended by the reviewer, for lines 154-156 we have changed the sentence to read “… whereas parental INS-1 and control lines had both an unmethylated and a methylated allele (**Fig 1D**) indicative of biparental inheritance of the PWS-IC imprint …”.

21. Line 33-34: because of the word “or”, this sentence is confusing and suggests that the PWS cell line has a deletion of only the paternal allele, when in fact it has deletions of both maternal and paternal alleles.

We have changed this sentence in the **Abstract** to reflect this point, to “silent, maternal (control) and active, paternal-allele (PWS)”.

22. For each sequencing chromatogram, it would be helpful to state whether the PCR product was sequenced directly or cloned first.

All sequences were derived from direct sequencing of PCR products unless otherwise specified.

23. Line 170: it is unclear why PWS line 3 gives a larger PCR product given that it is a larger deletion. Knowing which PCR primers were used might help.

The basis of the larger PCR product is summarized in lines 172-176 (pp 9-10) of the main manuscript and then in some detail on pages 10-11 of the **Supp. Info**, in the **S5D** **Fig** legend, because of insertion of a 233- or 234-bp fragment of *E. coli* DNA specifically as part of the DNA repair mechanism to generate that deletion. Hence, the PCR primers are irrelevant to the larger PCR product although all PCR primers are fully described in **S7 Table** in the **Supp. Info**. The basis of the larger deletion in PWS line 3 is likewise described in some detail on pages 10-11 of the **Supp. Info**, in the **S5D** **Fig** legend, because of the presence of a “segmental duplication” in the rodent genome at this location.

Note that we previously referred on page 11 for the legend to **Supp. Info S5D Fig** to work to be described elsewhere (manuscript in preparation) but removed this and corrected the statement to “… is part of an uncharacterized segmental duplication.” In fact, while this segmental duplication is uncharacterized in terms of both in the literature and in mouse genome sequence assemblies, we have performed such studies and an independent “manuscript in preparation” provides a detailed description of these segmental duplications, functional implications, and other details that go way beyond the scope of the present manuscript.

24. The eIF2a P/T ratios shown in Fig 6B don’t seem to agree with the values plotted in Fig 6E.

Note that the previous **Fig 6B,E** are now **Fig 6C,D** for the resubmitted manuscript (to refer to in the text in numerical and alphabetical order). The ratios reported immediately below the western blot panel (**Fig 6C**) are the direct ratio of eIF2α-pSer51 divided by total eIF2α whereas the data shown in the graph (**Fig 6D**) represent the *relative* ratios to the basal control condition as stated on the y-axis label and can be seen with the control DMSO samples having an average of 1.0. To clarify this point and confirm the data, we reanalyzed the ratio starting from densitometry measurements and added the eIF2α P/T ratios for the control samples for panel **Fig 6C** and have added raw data and analysis to the numerical data supplement (**S1 Data**).

Similarly, **Fig 6E** has been adjusted to show the ratio of ATF6-N/Total for both control and PWS cell lines in DMSO conditions. For **Fig 6F**, we have also reanalyzed and revised the ATF6-N ratio to now be N/Total rather than the former N/FL-UG as the latter had greater variance across samples; in addition, we have added the quantitative data for the DMSO (basal or control) conditions with ANOVA and Tukey’s post hoc statistics as for **Fig 6B** and **Fig 6D**. Given the minimal basal activation of ATF6-N and the benefits of comparing bands within gels, **Fig 6F** is a graphical representation of data from the 5 hr tunicamycin treatment (from the gel in **Fig 6E** combined with a second set of biological replicates). This data along with those for **S19 Fig** are included in the **S1 Data** supplemental file.

25. Fig 1C: What is mixed pop?

We now define this in the **Fig 1C** legend, stating “The deletion breakpoint is also detected in DNA from a heterogenous population of transfected cells (mixed pop.).”

26. Fig S9B: What is NTC?

NTC, no template control. This is now defined in the legend to **S9 Fig**.

27. Line 251: What is Snord109? It is not shown in Fig 4B or in Fig 1A.

Thank you to the reviewer for catching this typo, which has been corrected to *Snord64*, the fourth PWS-region snoRNA present in rodent genomes. Note that *SNORD109* is a fifth PWS-region snoRNA present in the human genome and many other mammals but does not exist in rodent genomes.

28. Fig S4A legend: typo. It should read “distal sgRNA79-1”

The reviewers were incredibly careful, as they indeed caught this typo, which has been corrected!

29. Fig S19A typo: the control 2, 1 hr time point seems like it should be 0.03 instead of 0.3

Note that this is now **S18A Fig** for the resubmitted manuscript. The reviewers have indeed caught a typo in the figure itself, as they are correct that the value should be 0.03 for the 1 hr timepoint for control line 2. This has been corrected on the figure.

Reviewed (and signed) by Michael Ailion and Chau Vuong

**Have all data underlying the figures and results presented in the manuscript been provided?**
Large-scale datasets should be made available via a public repository as described in the *PLOS Genetics* [data availability policy](https://urldefense.com/v3/__http:/journals.plos.org/plosgenetics/s/data-availability__;!!NHLzug!MjKsalXc1AiZryyaN-yQhkpAPjCPH3cvN1dlkfS6_i7HmL5s0BpODx2kCEhXjpHy6iiYat1x2LiUuM1Khgppsw$), and numerical data that underlies graphs or summary statistics should be provided in spreadsheet form as supporting information.

Reviewer #1: Yes

Reviewer #2: Yes

These “Yes” responses highlight that we have submitted two sets of RNA-seq data and three sets of proteomics data to database repositories and have included extensive data in **Supp. Info** figures and tables.

Reviewer #3: **No:** Unpooled source data for figures is not available. Raw data for insulin secretion experiments is not available. Though transcriptomic and proteomic data have been submitted to public data repositories, I was not able to access the proteomic data (required a password) and it is unclear to me whether only the original RNAseq data is available or processed data too. It would be very helpful to have supplementary tables of these data with lists of proteins or genes and their expression values (as is common in many papers).

The “No” response reflects in part that we had not with the initial submission included all the numerical data and statistical analyses, for which we have now rectified this as per *PLoS Genetics* journal policy with an **S1 Data** (Excel file) submission containing tabs for all graphical elements in the main manuscript and the **Supp. Info**. The comments on the RNA-seq data somehow fails to recognize that we have presented all significant differentially expressed RNAs in **S1 Table**, **S2 Table**, **S3 Table**, **S4 Table**, and **S5 Table**. In addition, we have submitted and made publicly available since June 01, 2022, the raw .fastq files, transgene and unannotated feature genomic annotation .fa and .gtf files, a comprehensive summary metadata file, and unfiltered complete normalized gene expression lists from a STAR-htseq-deseq2 or RSEM pipeline (code provided on public git repository) to the NCBI GEO data repository. While the proteomics data at the time of manuscript review was password protected, our understanding from the repository was that a password was available for official journal reviewers, and all three sets of proteomics data will be released at the time of publication.

**List of Additional Changes in the Manuscript PGENETICS-D-22-00722 (article for *PLoS Genetics*)**

- All changes in the main manuscript are clearly visible using “Track Changes” within the “Marked” version of the text that is submitted for this revision (the “Marked” version includes the six **Figures** in the main manuscript). Please note that in all references below to changes in text “line numbers”, that these refer to line numbers within the fully revised “Clean” main manuscript text (removing the “Track Changes” as well as the **Figures**, with only the Figure legends in the “Clean” version). Finally, a small subset of “last-minute” changes to finalize formatting for *PLoS Genetics* were not included in the “Marked” version but are all within the “Clean” version, limited to changing “**Fig. n**” to “**Fig n**”, “**Fig. Sn**” to “**Sn Fig**”, and “**Table Sn**” to “**Sn Table**”, where n=1-6 for figures, n=1-19 for **Supp Info** figures, and n=1-9 for **Supp. Info** Tables.
- We have removed the separate **Supp. Info** PDF and rather now include all **Supp. Info** **Figure Legends** and **Supp. Info Table Legends** in the main manuscript, after the **References** and **Figure Legends** sections, and have uploaded as separate JPEG files all 19 **Supp. Info Figures** and as Excel files all 9 **Supp. Info Tables**.
- All six figures in the main manuscript have been modified as follows:
- **Fig 1B** had the blue background lightened, for improved visualization as well as printing.
- **Fig 2** was redrawn to present each individual data point for each cell line, as per Reviewer #3, Q10.
- **Fig 3A-D** were redrawn as specified by Reviewer #2, minor issue 1.
- **Fig 4** was redrawn, for **Fig 4A** to increase the size of lettering and improve representation of labeled RNAs, to move some of the less important panels to a complementary **Supp. Info** figure (now **S12 Fig**), and to reanalyze the normalization for all RT-ddPCR graphs (**Fig. 4C-F**) in combination with presenting all numerical data and statistics in **S1 Data**. All results were validated, for each of **Fig 4C-F** and for **S12 Fig**, with the exception that the RT-ddPCR data for each of the rat endogenous *Ins1* and *Ins2* genes is statistically significant due to removing one data point from each dataset that was more than 2 standard deviations outside the mean (see point below).
- **Fig 5** was changed only by the lettering to pair each western blot with its corresponding graphical quantitation (i.e., **A-B**, **C-D**, **E-F**), also resulting in describing **A** through **F** in order in the main text.
- **Fig 6** was changed in part by the lettering to pair each western blot with its corresponding graphical quantitation (i.e., **A-B**, **C-D**, **E-F**), also resulting in describing **A** through **F** in order in the main text.
- **Fig 6C,D** was changed as described in detail in the response to reviewer # 3, Q24, see above.
- **Fig 6E,F** was changed as described in detail in the response to reviewer # 3, Q24, see above.
- All six figure legends have been modified in accordance with these changes in the figures, and all changes in the legend text are visible by Track Changes in the “Marked” version of the manuscript.
- The new RT-ddPCR data analysis for rat *Ins1* and *Ins2* genes altered a minor conclusion from the study and indicated that both endogenous insulin genes were slightly downregulated at the mRNA level in accordance with other genes encoding secreted peptides (i.e., *Iapp*, *Npy*, and transgenic mouse *Ins2* and *Ins2*-*mCherry*). This necessitated minor changes in the **Abstract** text (lines 39-41) and the **Discussion** (lines 425-427).
- For all **Supp. Info** figures we replaced the previous use of lower quality JPEG images within a Word and then PDF file with direct upload of higher resolution and quality JPEG images (in RGB format), as noted also in the response to Q18 from Reviewer #3. Other changes in **Supp. Info** (other than in response to reviewer comments) included:
- Inclusion of **S1 Data**.
- The previous **S16 Fig** was moved to **S12 Fig**, was expanded by adding additional panels **A**, **C**, and **E** (all originally in **Fig 4**), and – as noted above for the complementary **Fig 4** -- underwent reanalysis of the normalization for all RT-ddPCR graphs (**S12B-E Fig**) in combination with presenting all numerical data and statistics in **S1 Data**. All results were validated, although the data for *Robo2* and *Id4* was now significantly different between PWS and control groups (now in **S12B Fig**). Finally, we added RT-ddPCR data for *Pcsk2* (not previously included) to **S12E Fig** to pair with the *Pcsk1* data.
- The latter change led to renumbering of **S13 Fig**, **S14 Fig**, and **S15 Fig**, from **S12**-**S14 Fig**.
- Additional description was included in the legend for **S17 Fig**, as noted above to Q15 of Reviewer #3.
- The previous **S18 Fig** (confocal images) was deleted, as noted above to Q16 of Reviewer #3. This led to renumbering of **S18 Fig** and **S19 Fig**, from **S19**-**S20 Fig**.
- A correction for the renumbered **S19A Fig** is described above to Q29 of Reviewer #3.
- The **Supp. Info** reference list was incorporated into the main references (added 7 references), with three references added to the legend of **S12 Fig**, two in the **S15 Fig** legend, and two references added to the expanded **S17 Fig** legend.
- As noted in the Cover letter and at the end of Reviewer #3 responses above, we now include as per *PLoS Genetics* journal policy an **S1 Data** (Excel file) submission, with all Numerical Data and Statistics.
- The main manuscript reference list was expanded slightly due to changes in the text, including 6 new reference citations, for Chen et al. 2022 (ref. 33); Adams et al. 2018 (ref. 55); Bradley et al. 2005 (ref. 95); Szczerbinska et al. 2022 (ref. 69); Szabat et al. 2016 (ref. 96); and Aklan et al. 2020 (ref. 107). These were based on newly published work, our updated data analysis, and literature scholarship, including with the addition of an important new paragraph in the “Mechanisms” section on pp 24-25 (lines 506-523) of the **Discussion**.
- In the **Introduction**, we added a brief note of further evidence supporting one of the PWS-imprinted genes being dysregulated in the human pancreatic islet (ref. 33, line 100) and changed that sentence accordingly.
- In the **Results**, changes in text due to reanalysis of numerical data and statistics (as in **S1 Data**), are included in line 302; lines 306-311; lines 314-318; and lines 327-328. Additionally, a new sentence in lines 290-292 reflected comment on additional biological pathways identified by the gene ontology analysis (**Fig 4B**) but not previously noted.
- In the **Discussion**, changes in text are included in lines 425-429 (as noted above, relating to mRNA levels of endogenous insulin genes); line 442 (to state “contributes to” rather than “directly results in”, since the former is strongly supported by our data whereas we do not have proof of the latter); lines 486-487 (change in subsection title, to broaden the scope of discussion on potential mechanisms to include alternative possibilities); lines 494-495 (to specify that it is unknown as to the level of transcription or RNA metabolism that the putative GRN is controlled); lines 508-525 (to include a new paragraph on an alternate explanation for decreased ER chaperone levels in PWS cells in association with decreased insulin levels, and how the literature provides support that this hypothesis appears unlikely); and line 526 (to simplify the statement).
- In the **Methods**, changes in text are based on revised analyses of data (as part of adding an **S1 Data** file) as well as in response to reviewer questions (all described above) and are included in lines 659-661; lines 669-671; line 677; line 679; lines 681-688; lines 736-740; line 753 (section on “Confocal microscopy” deleted); and lines 755-756. We also removed “Costa RA et al., manuscript in preparation” from line 594, replacing it with a previously cited literature citation (ref. 42).
- In the **Data Statement**, changes in text include updated Proteomics database identifier information in lines 793-796; and inclusion of a statement on addition of an **S1 Data** file in lines 797-798. The previous **Acknowledgements** section was split into **Funding** and **Acknowledgements** sections on pg. 38, as per *PLoS Genetics* policy. Minor changes were also made in **Acknowledgements**, lines 809-810 to update the support from the Penn RIA/Biomarkers Core, and in the Funding section, lines 801-802 to update from singular to plural for grant support from FPWR.
